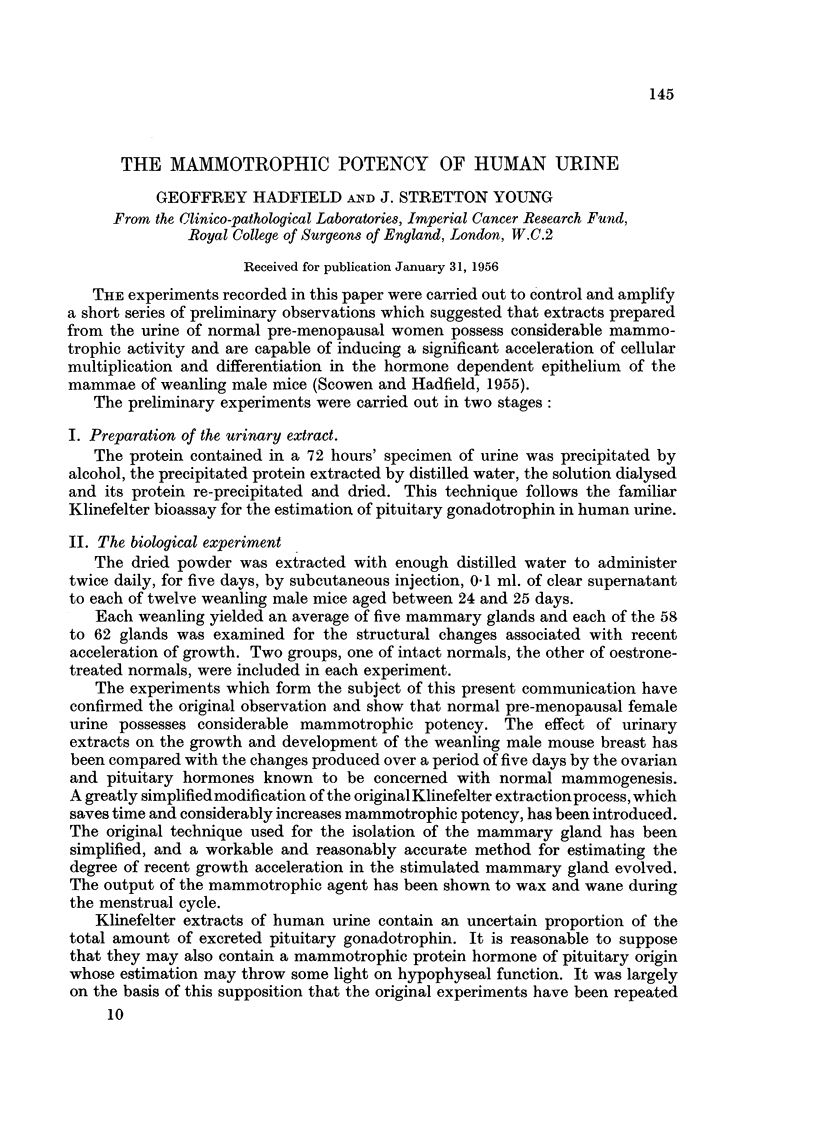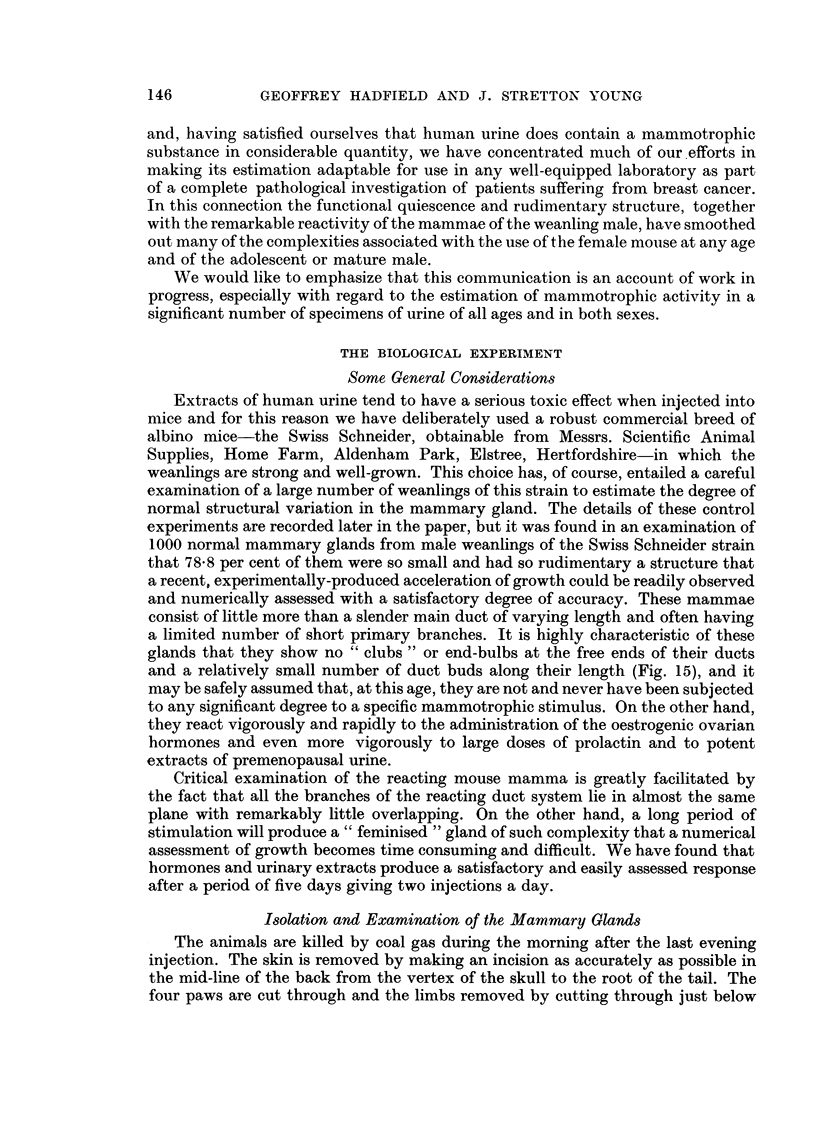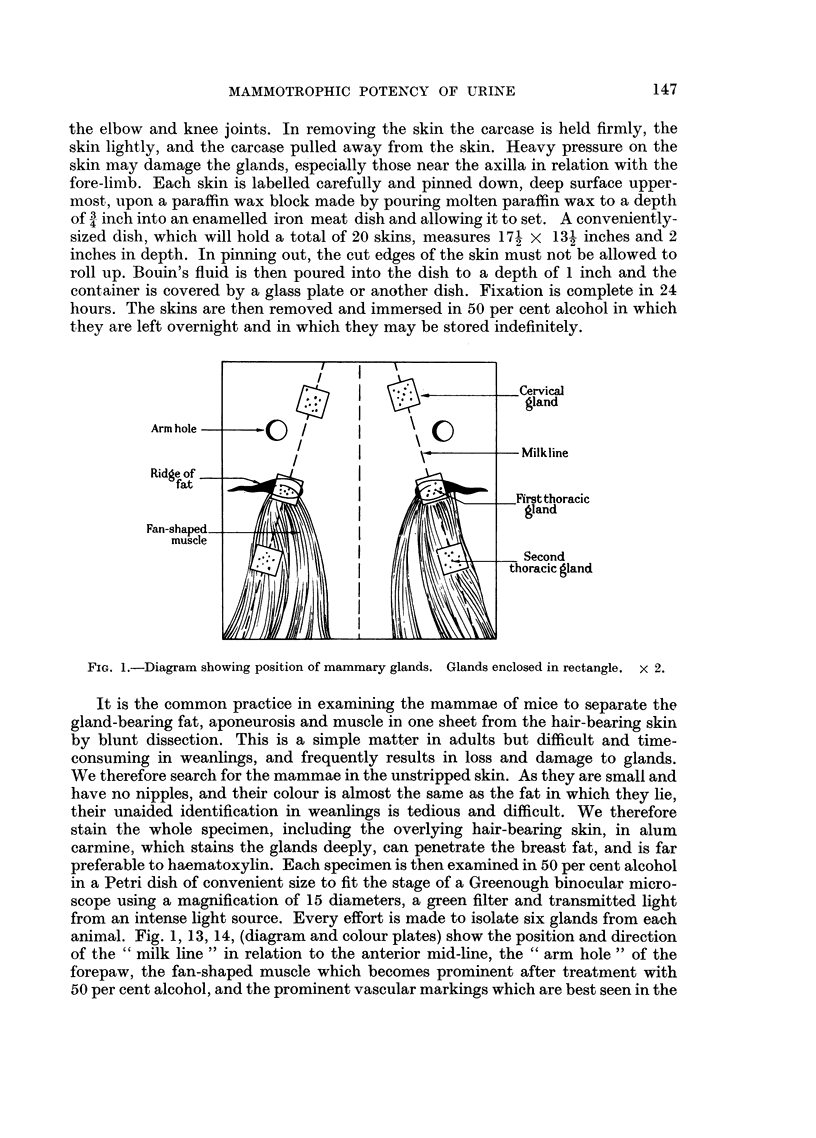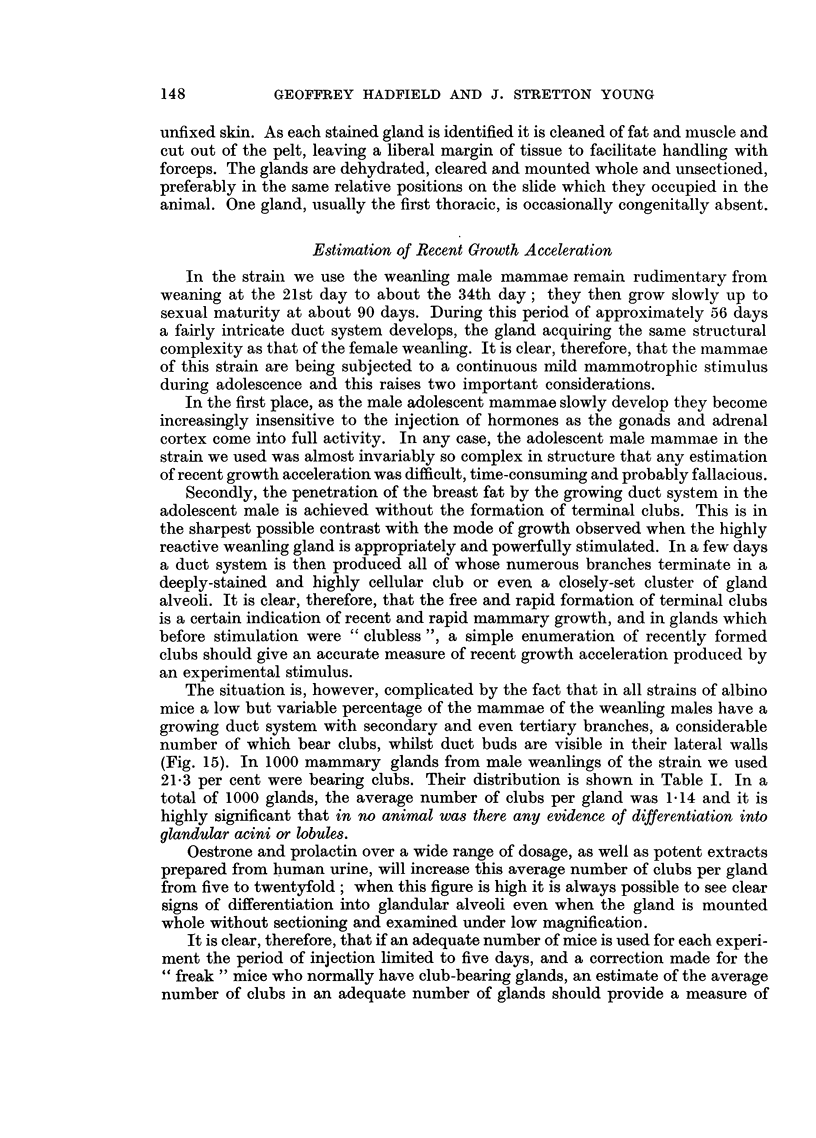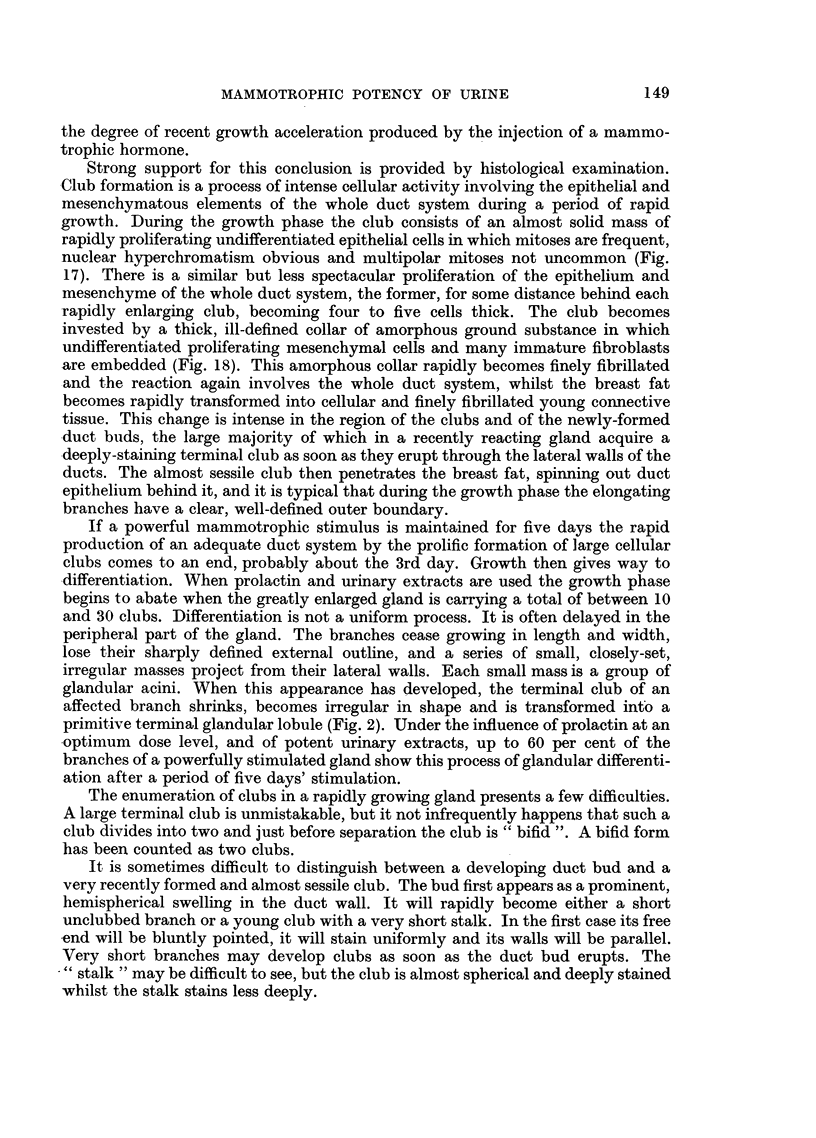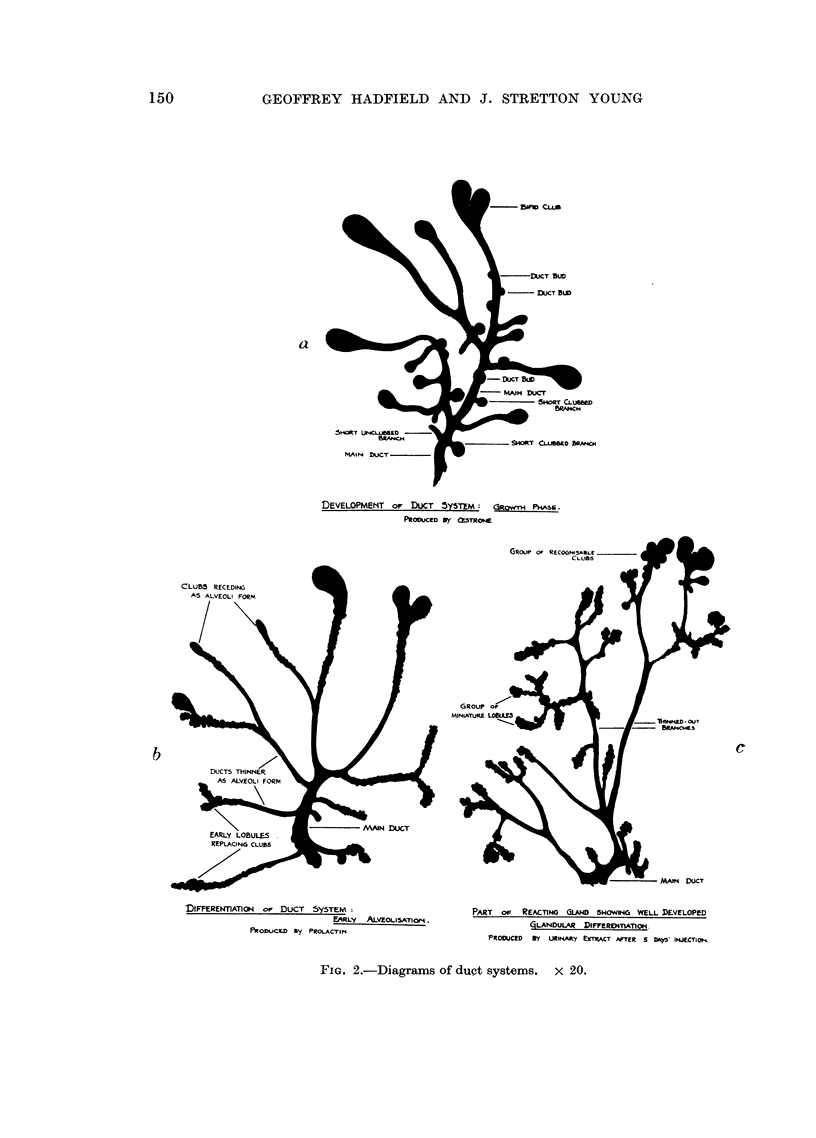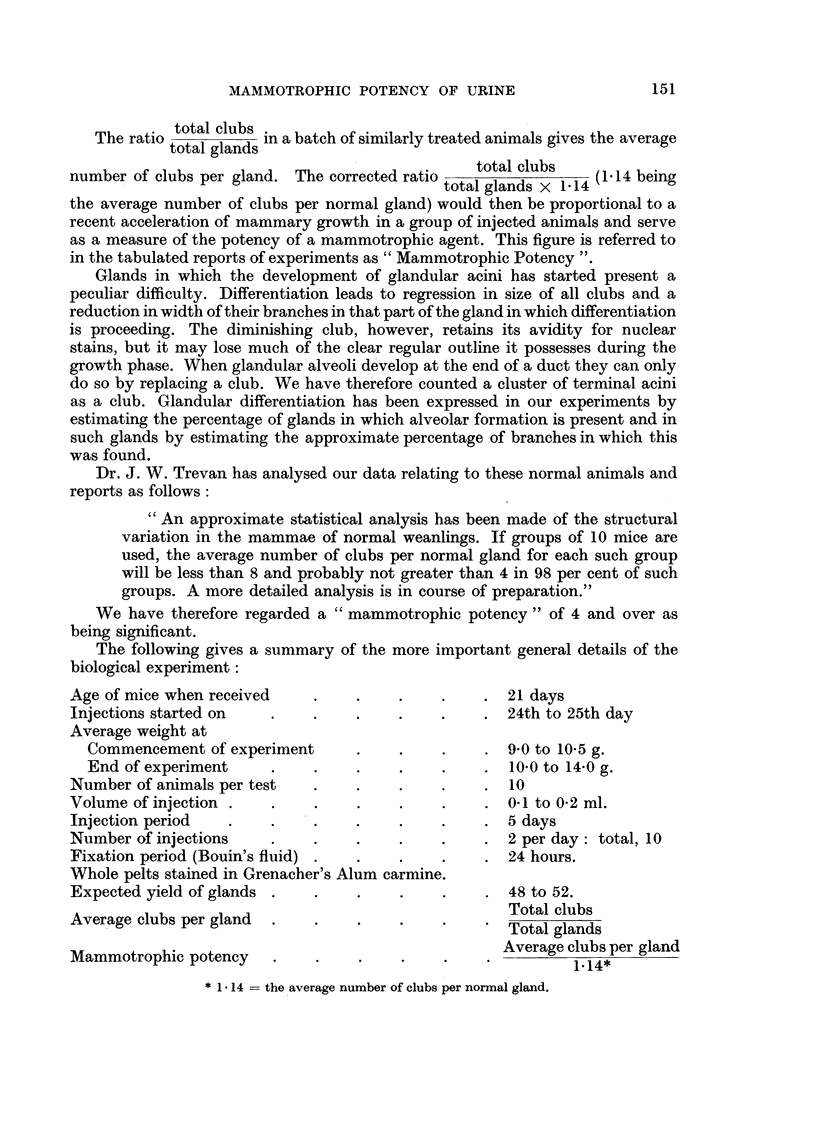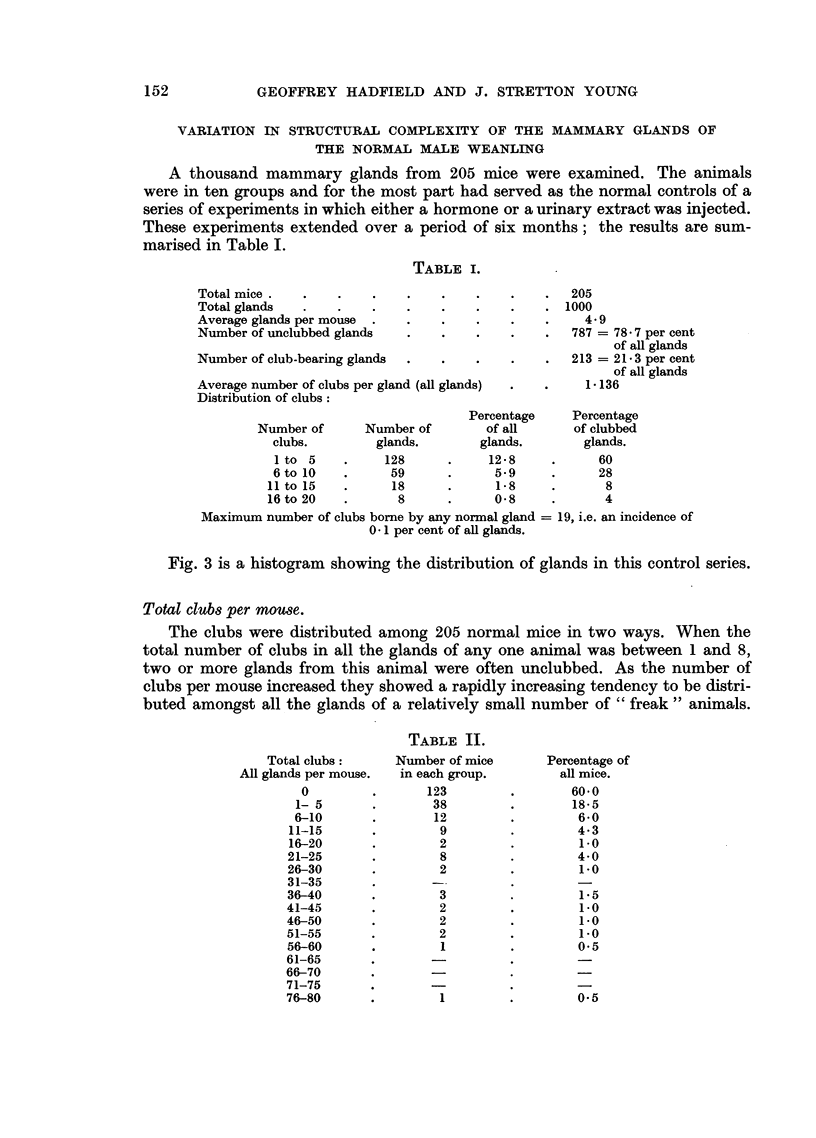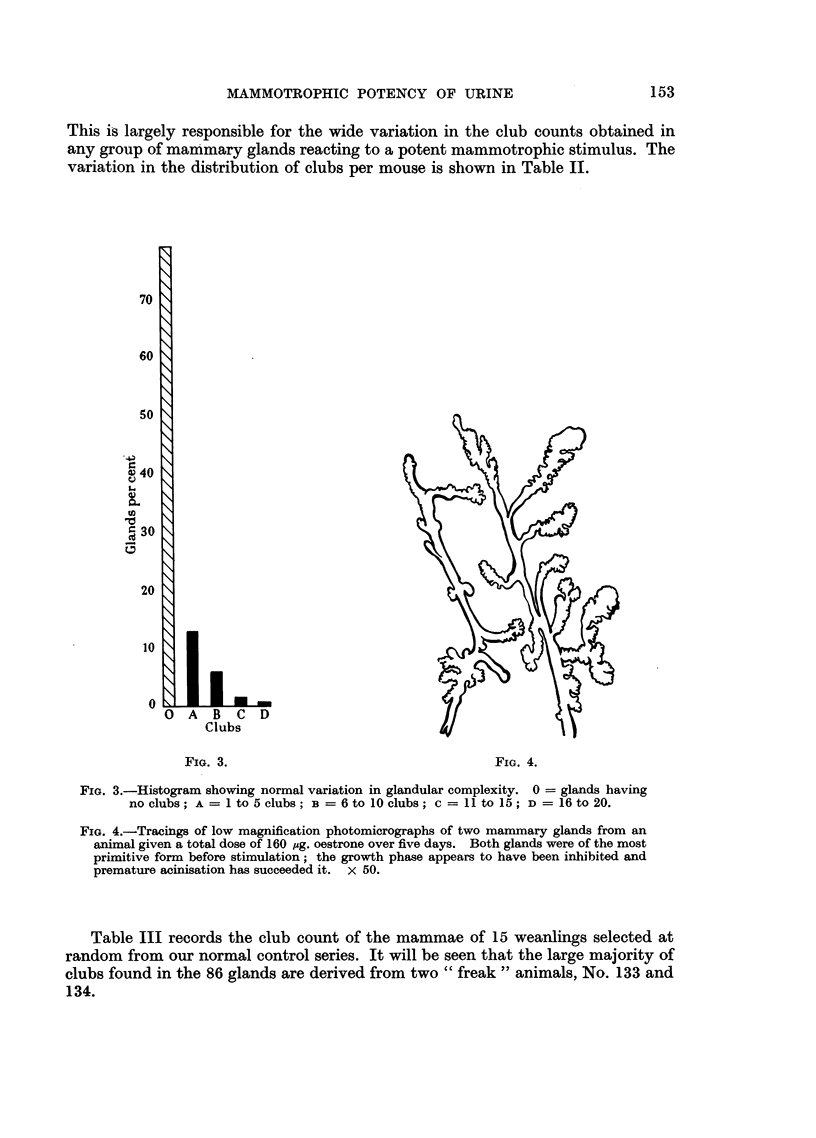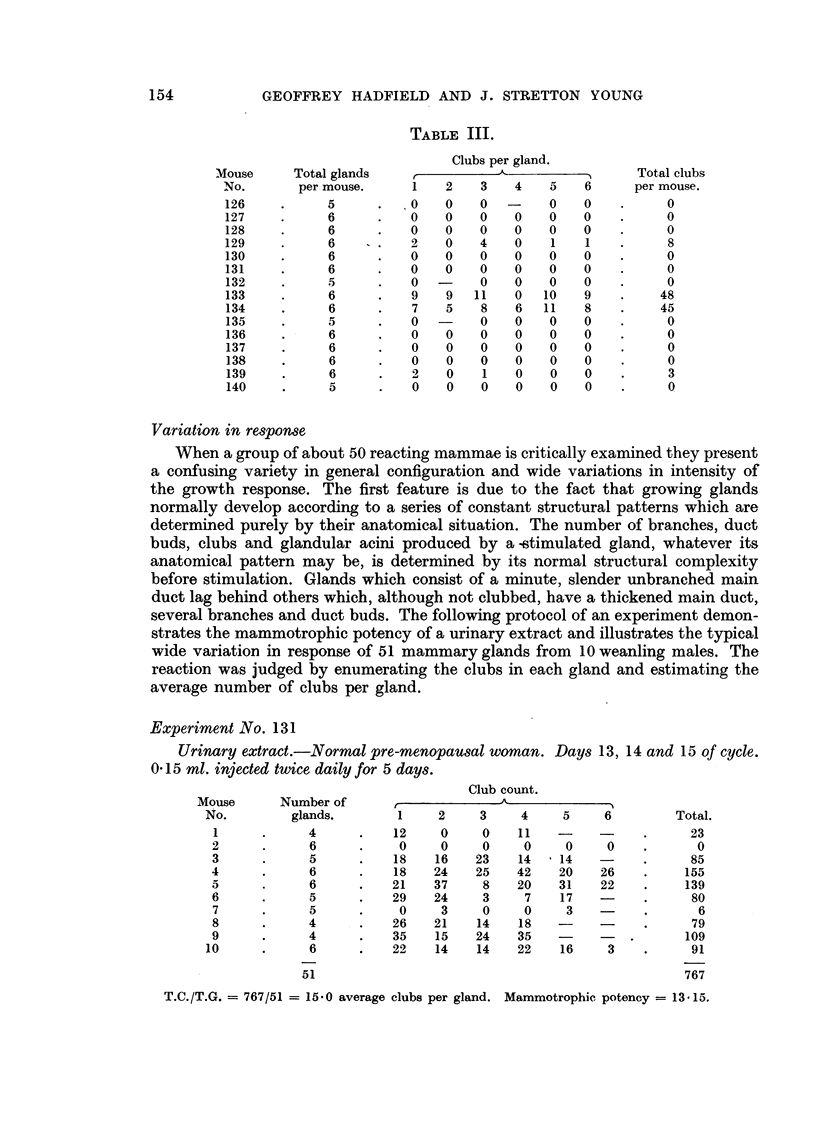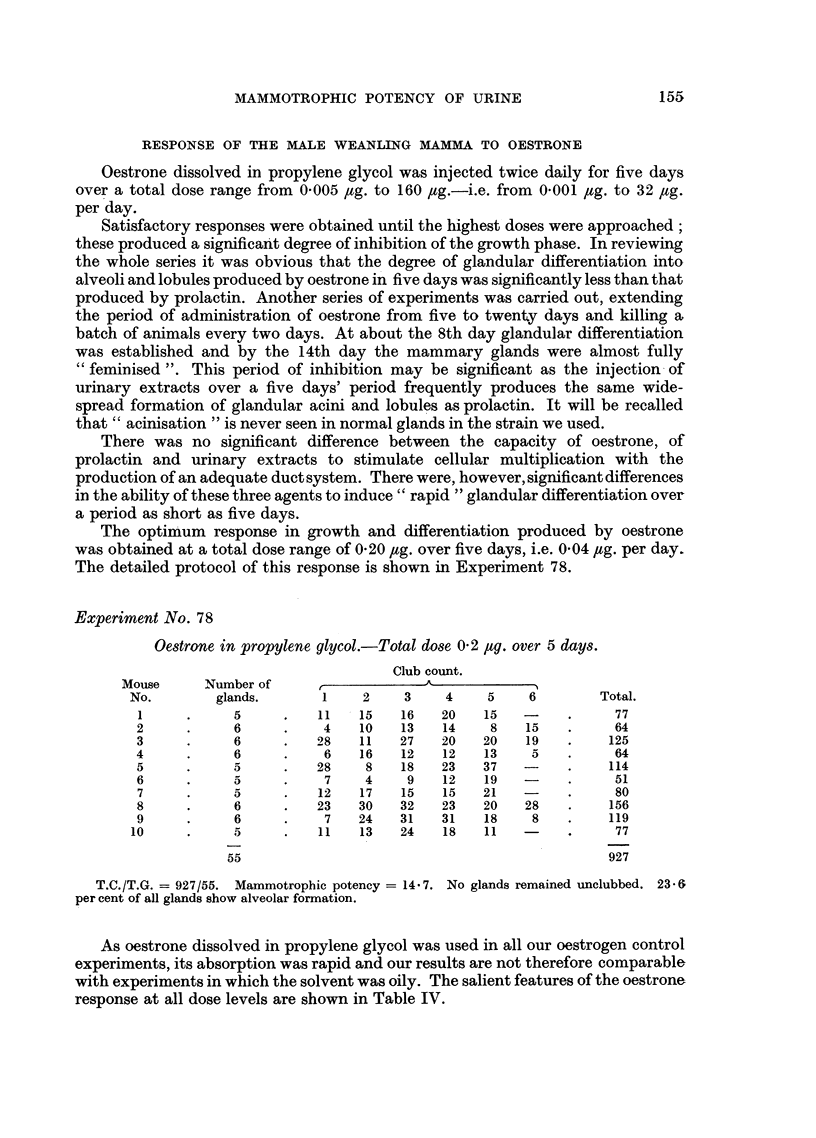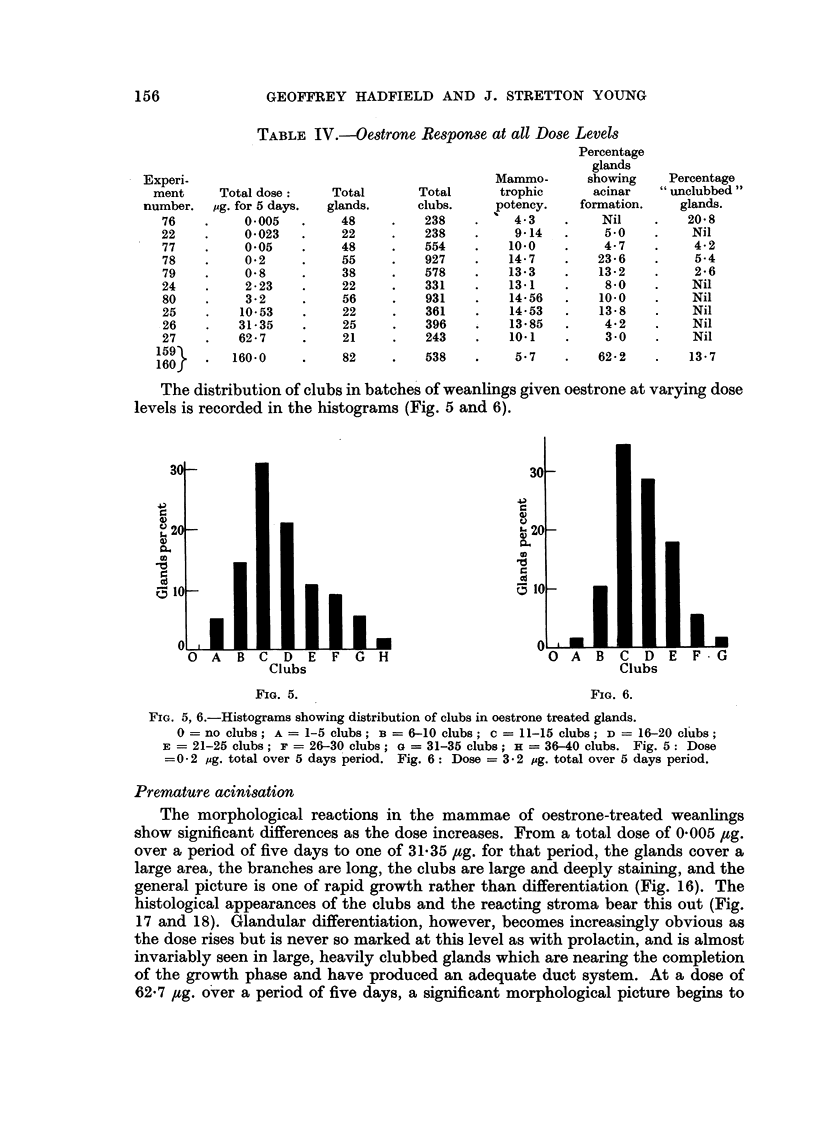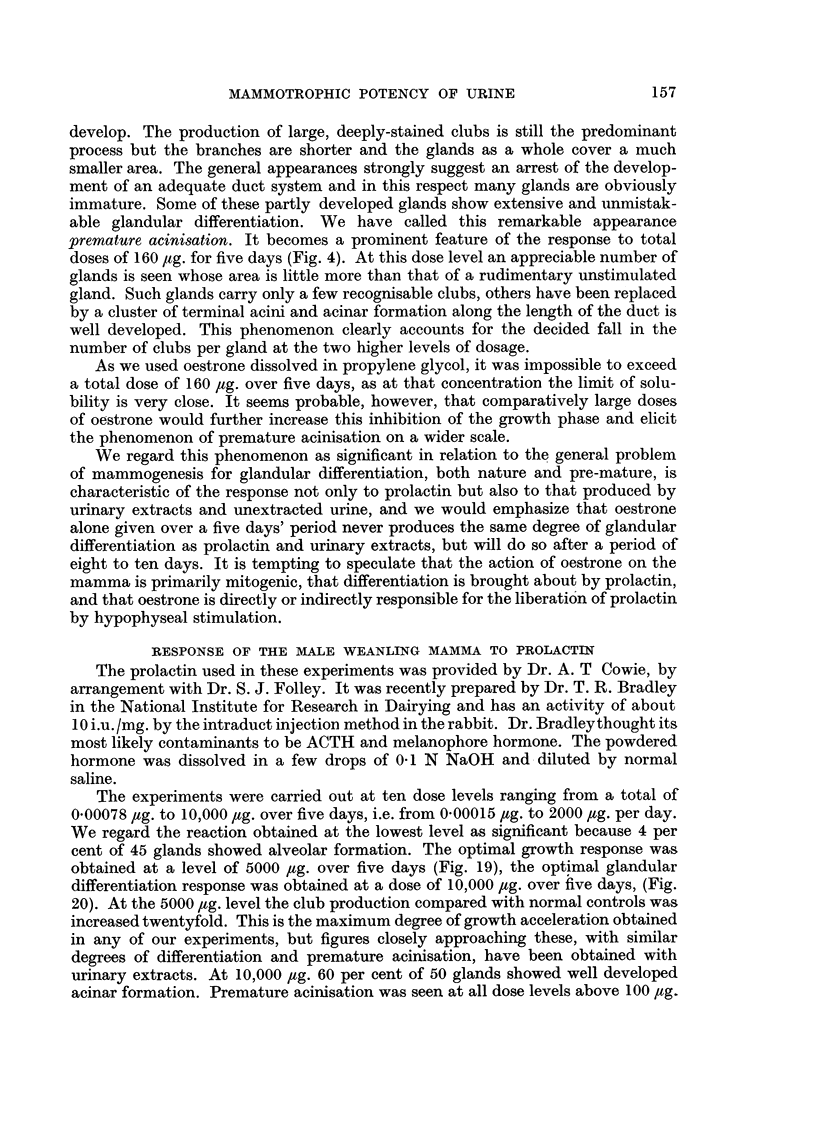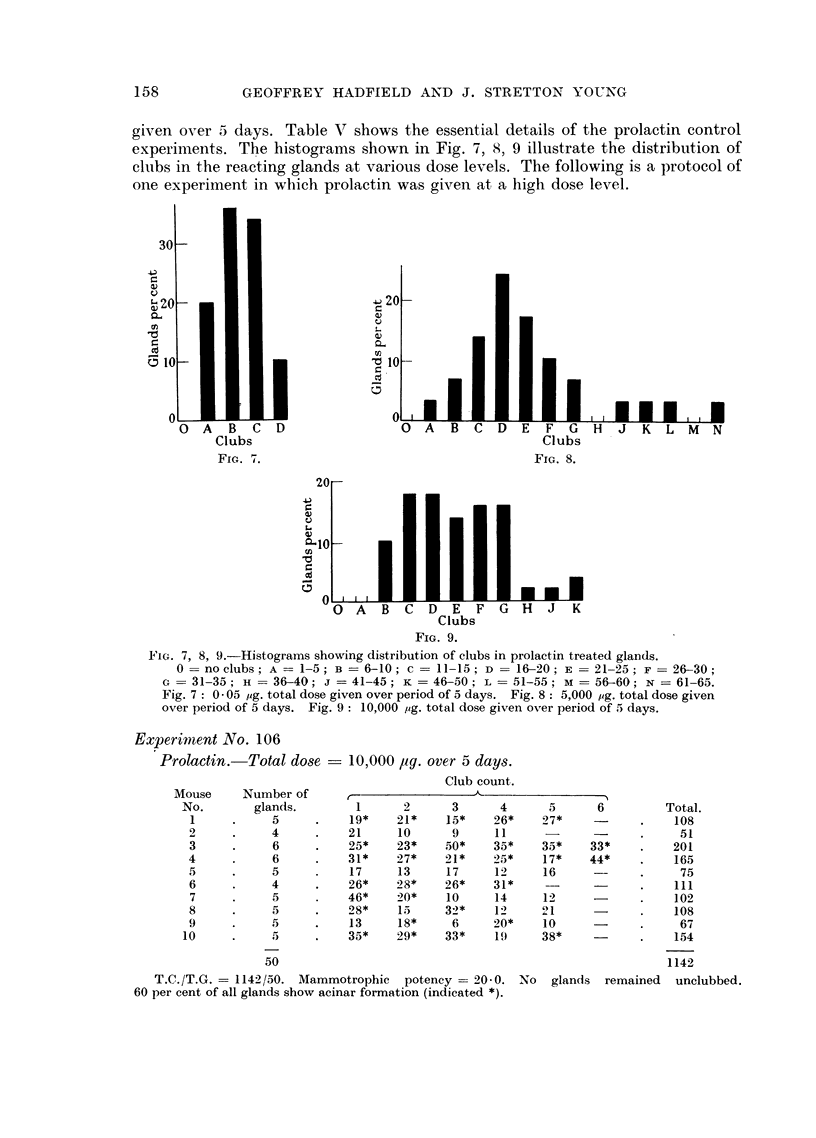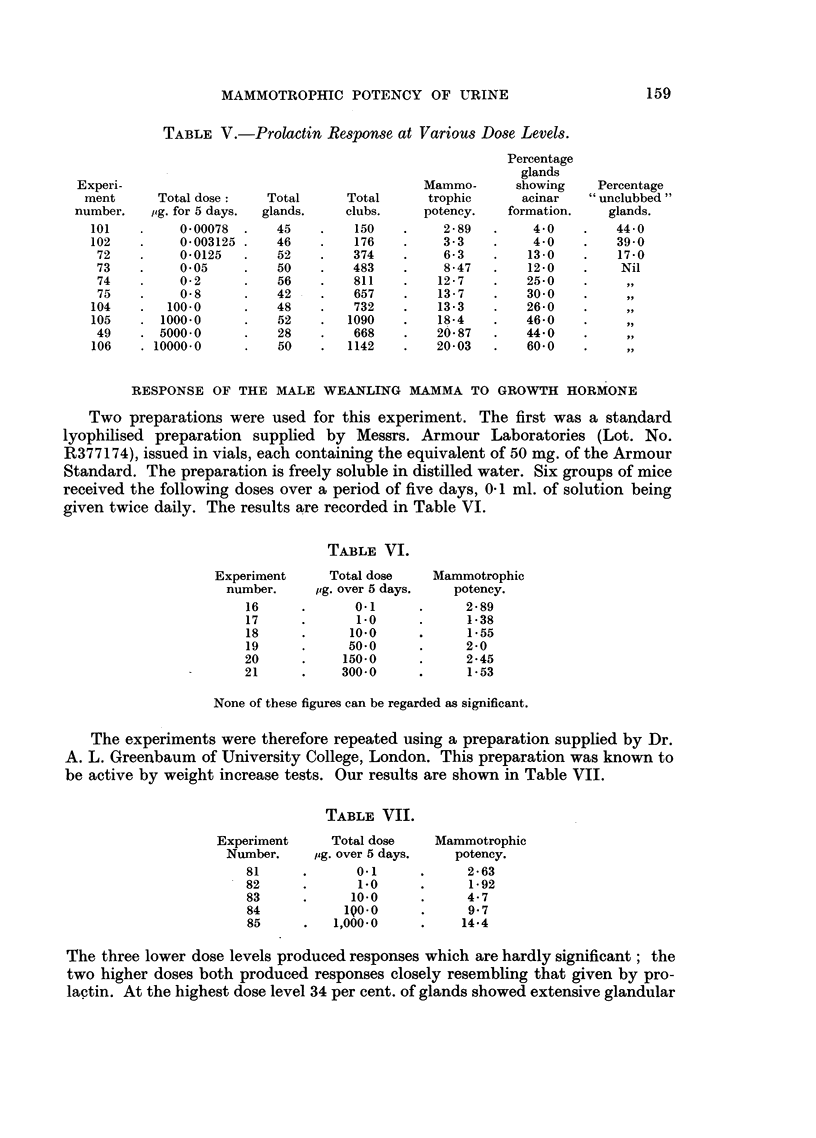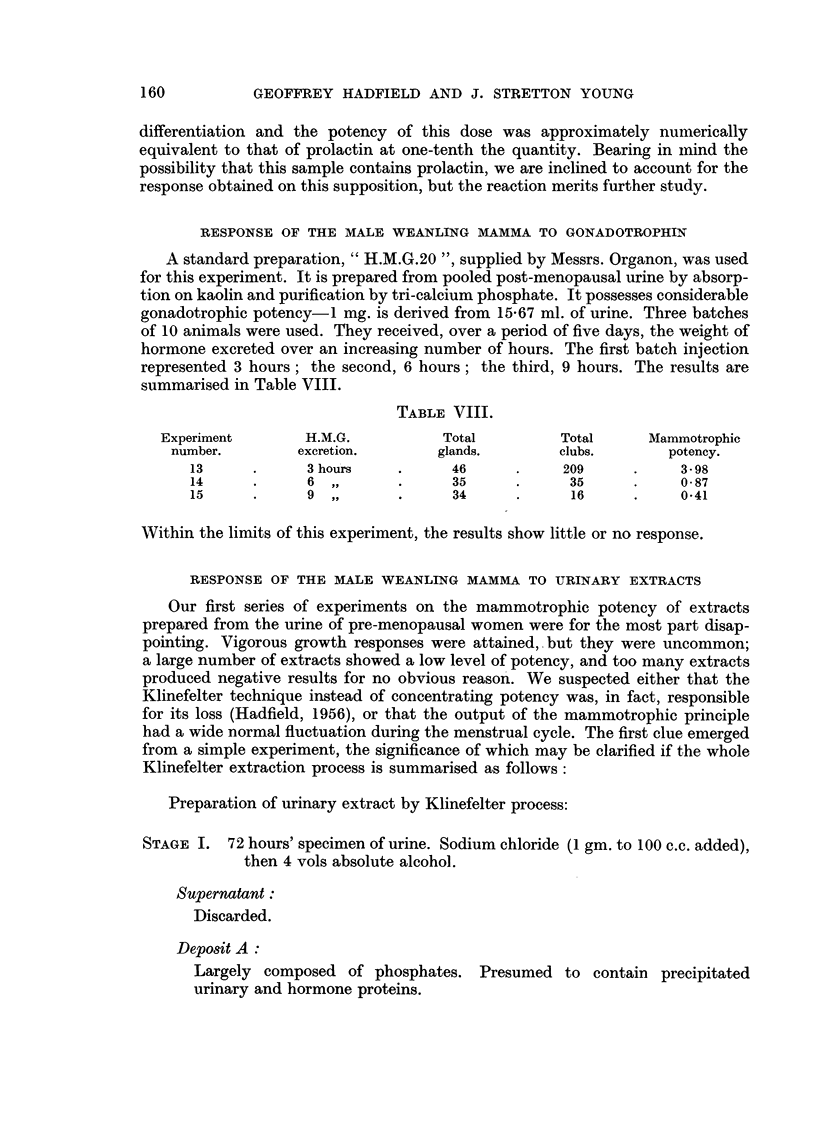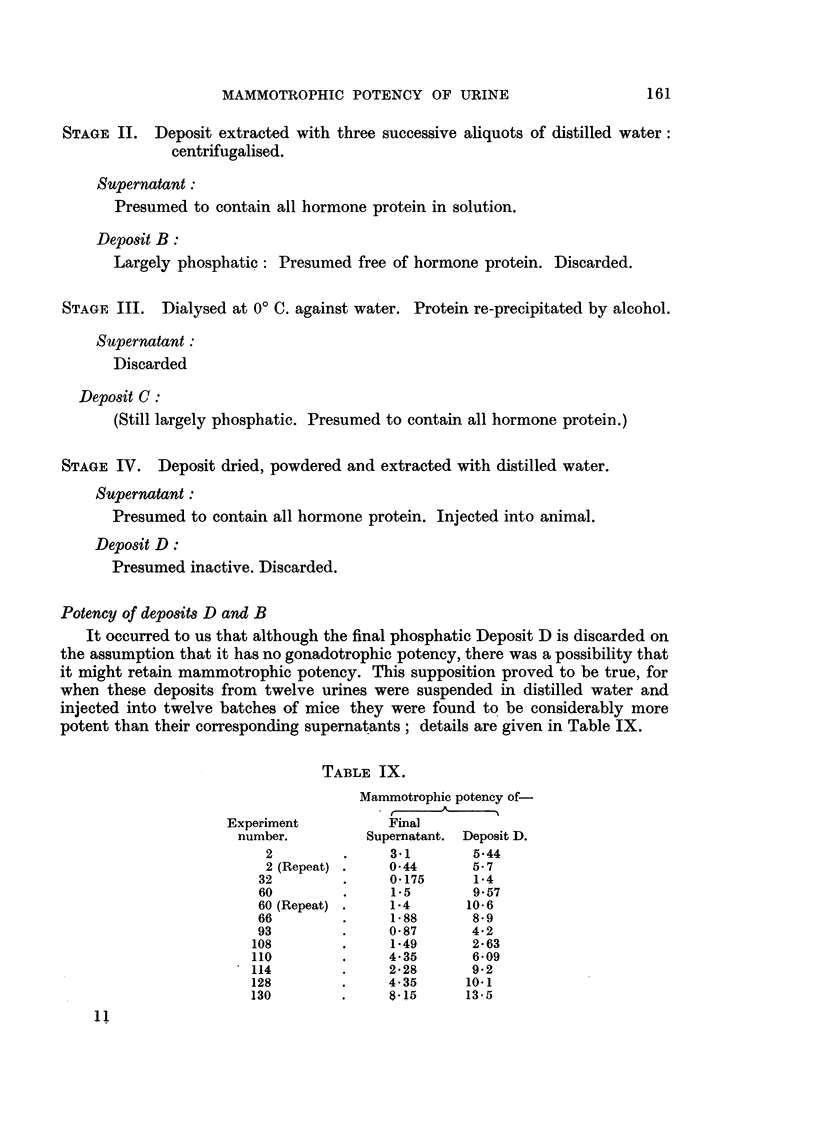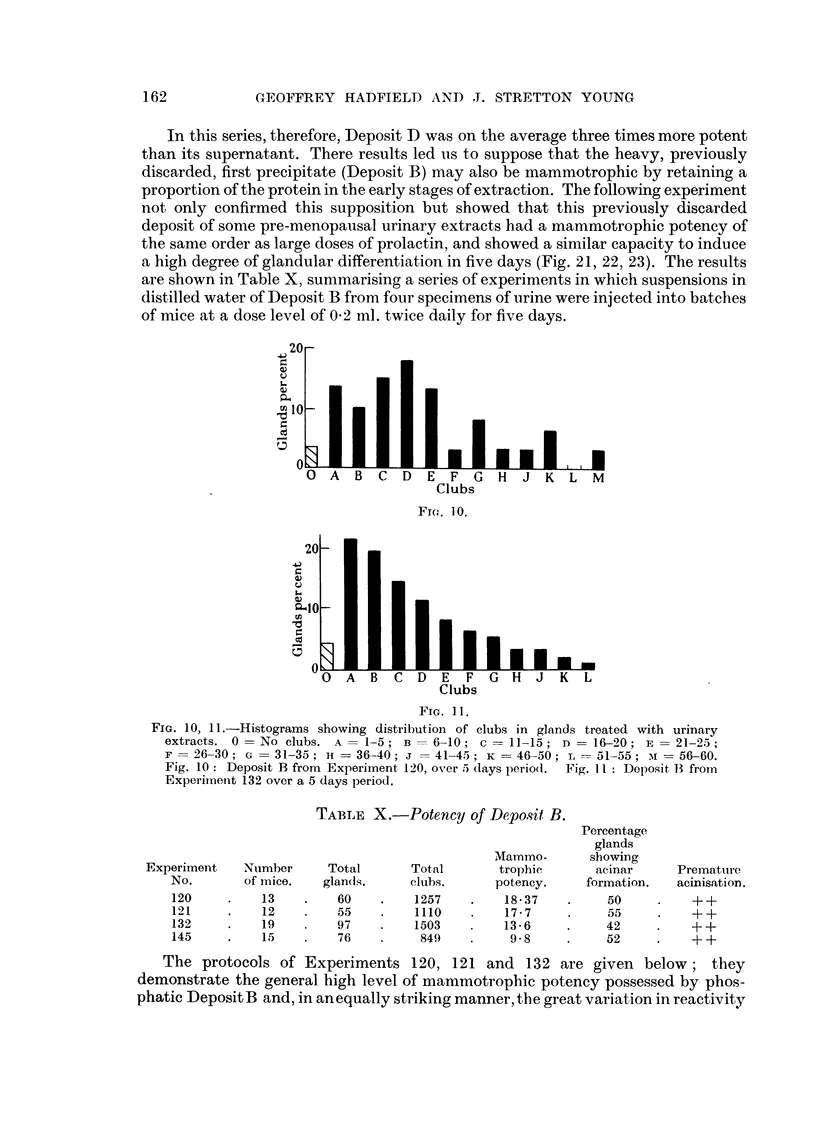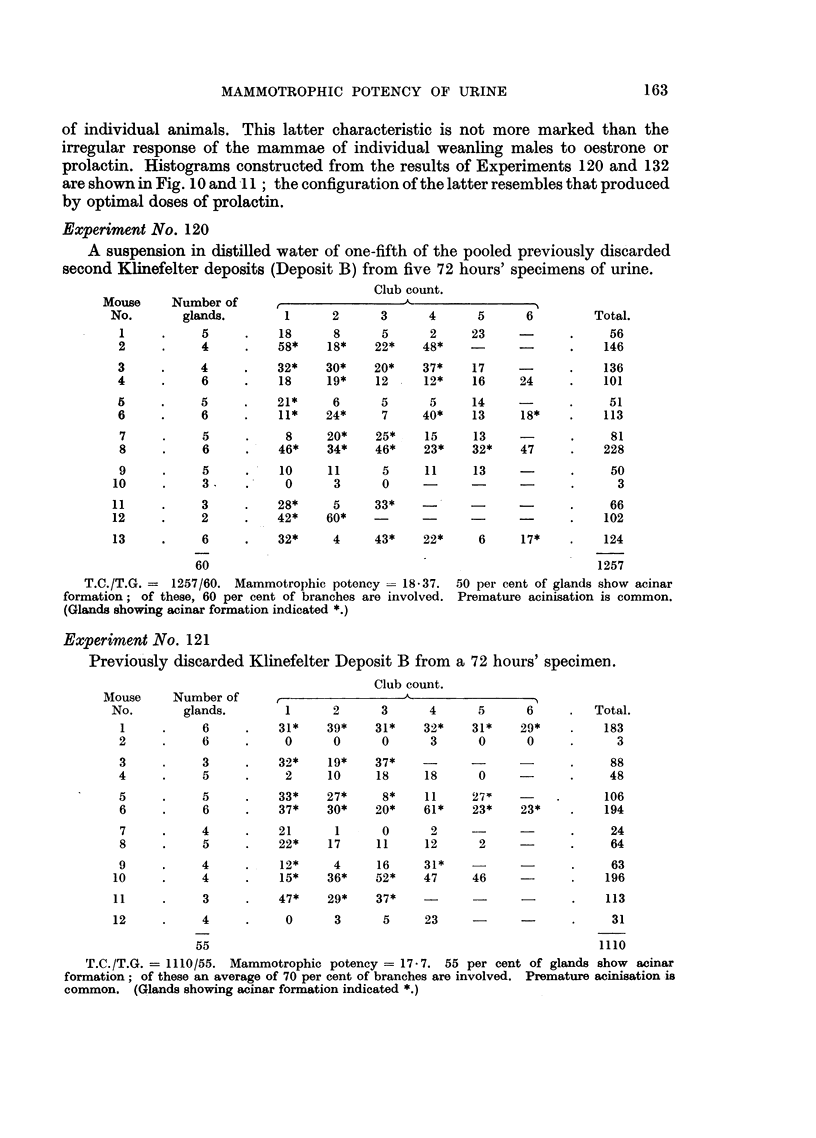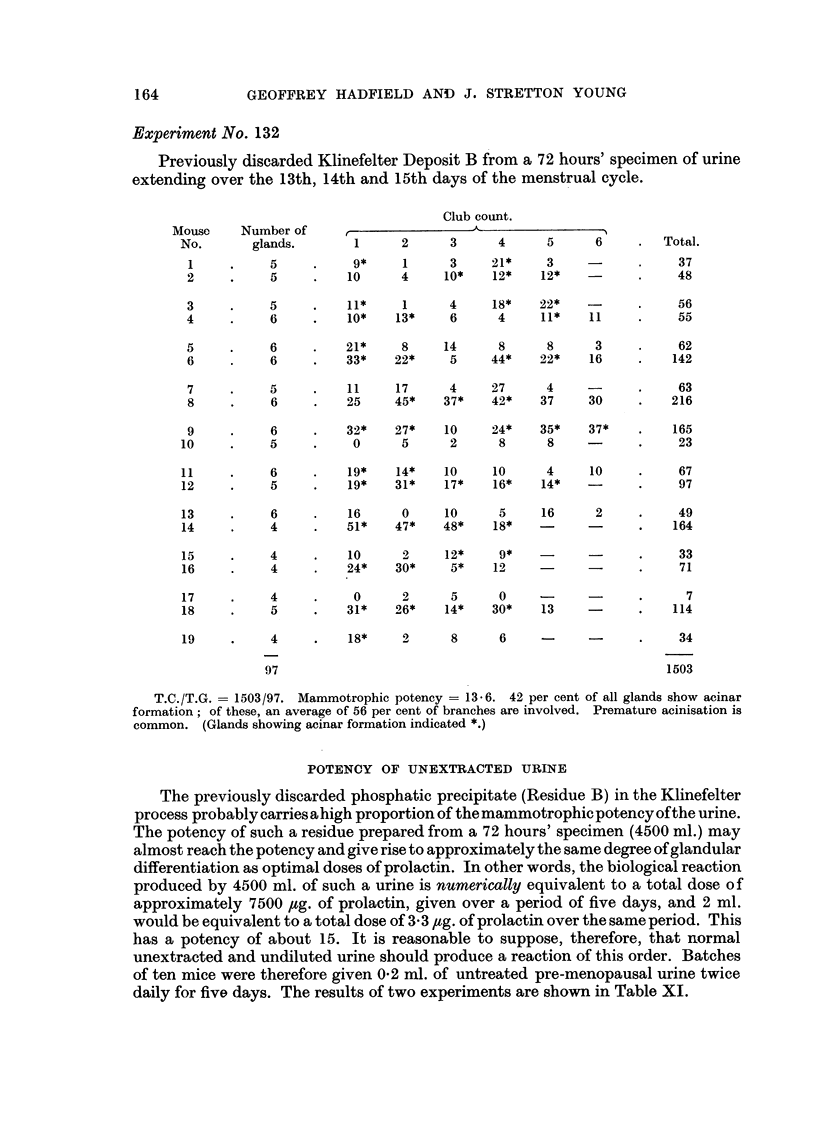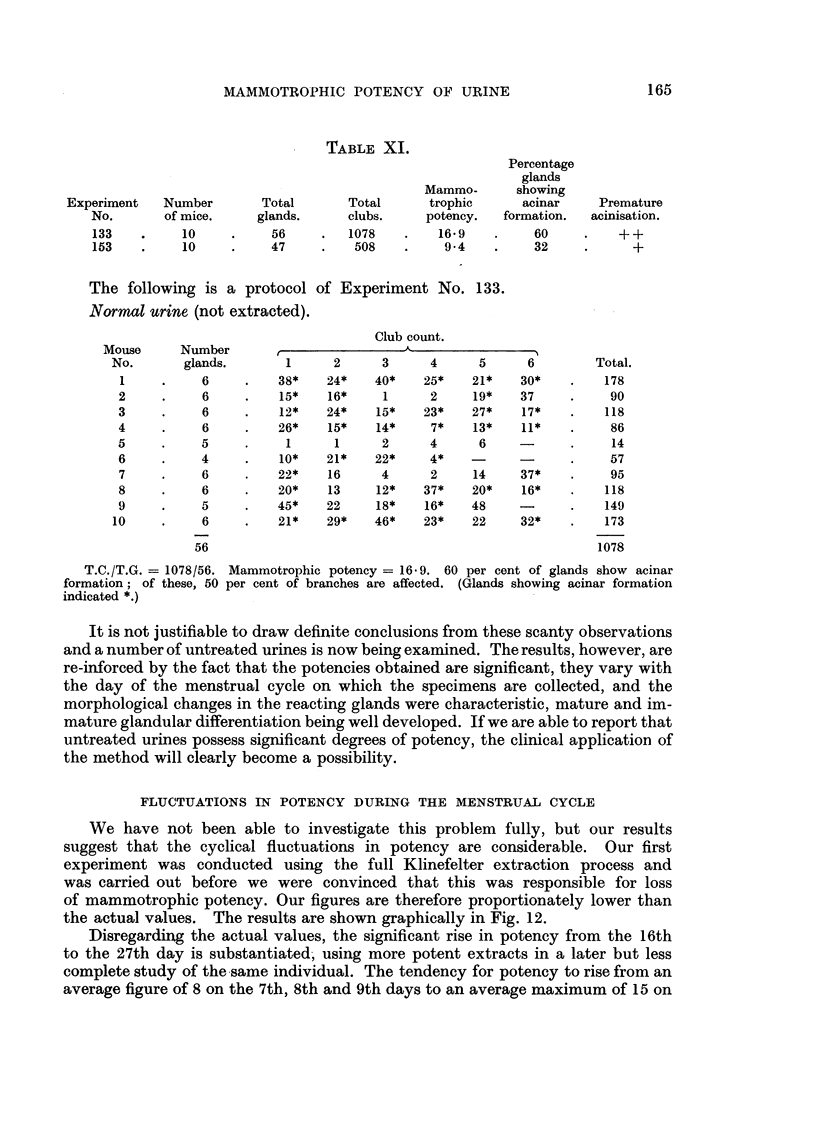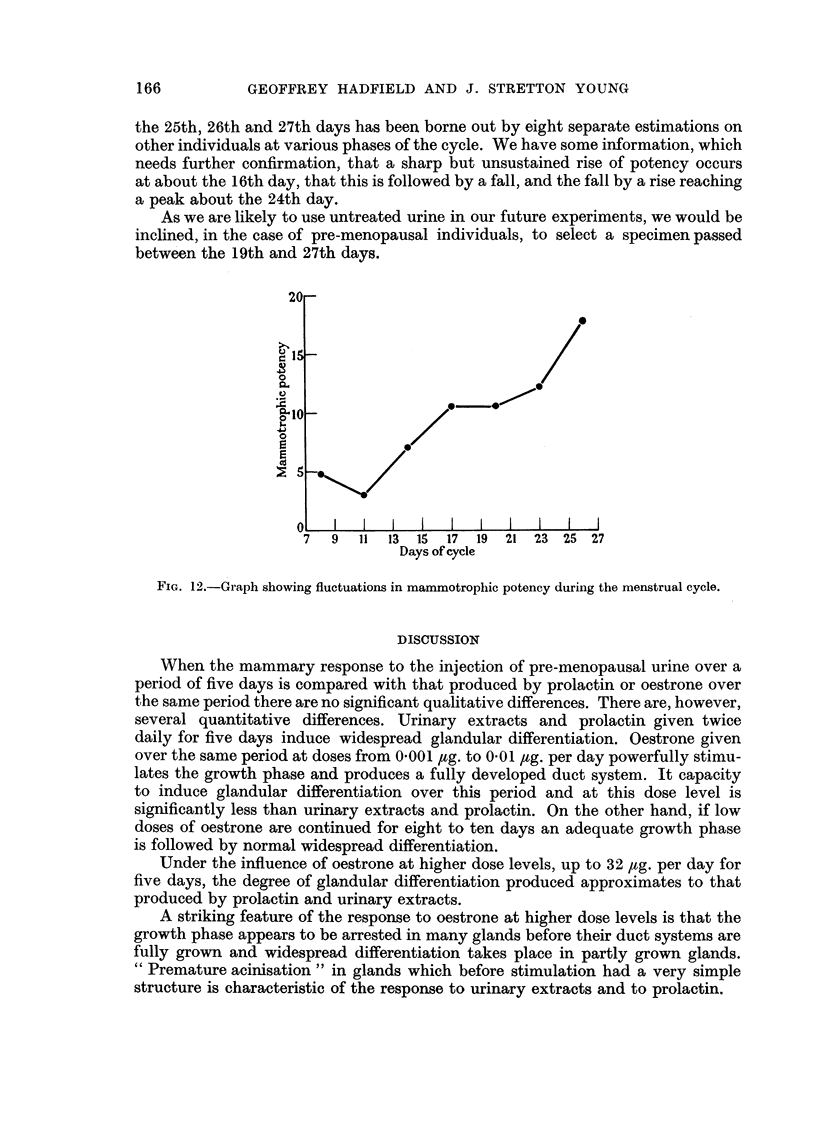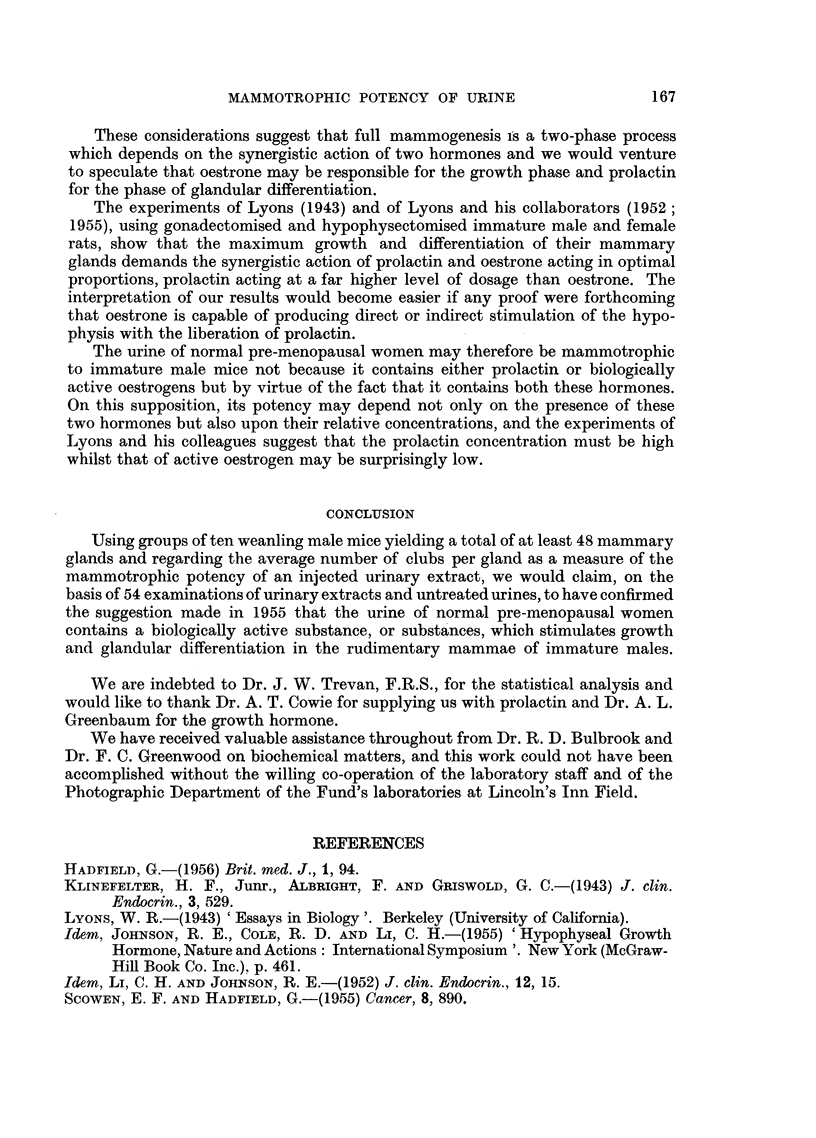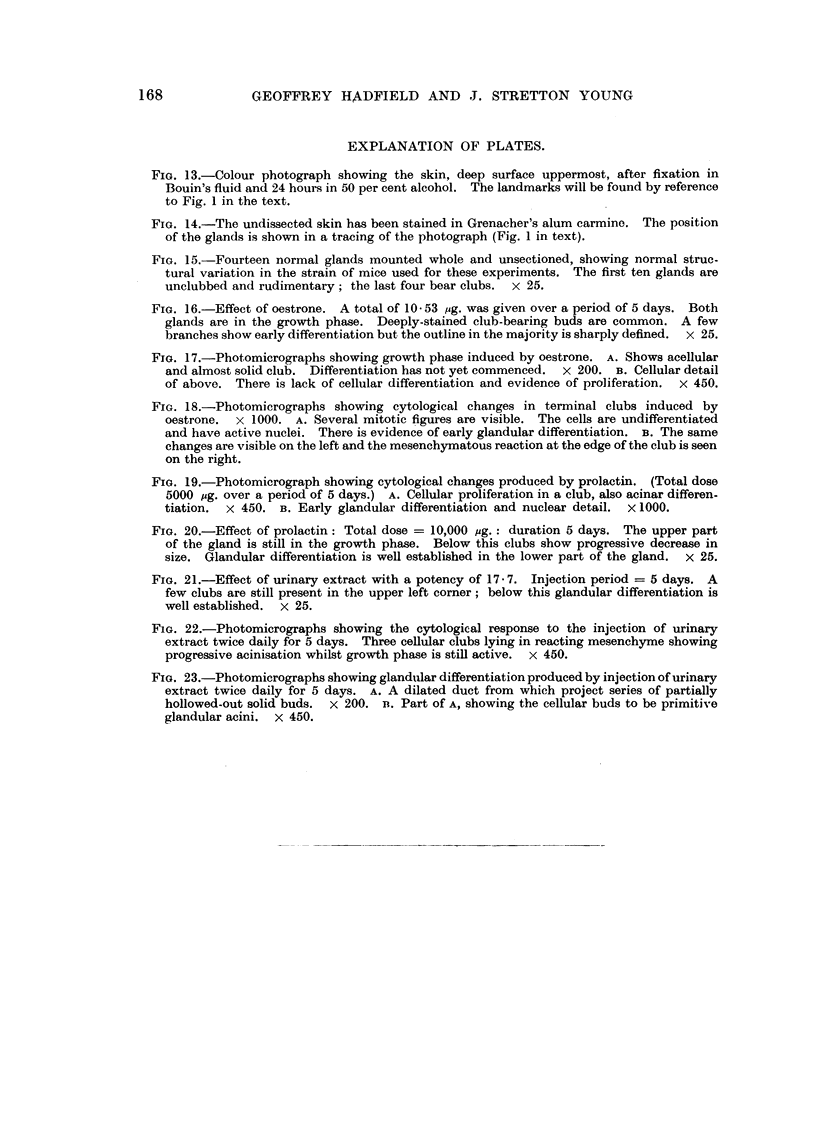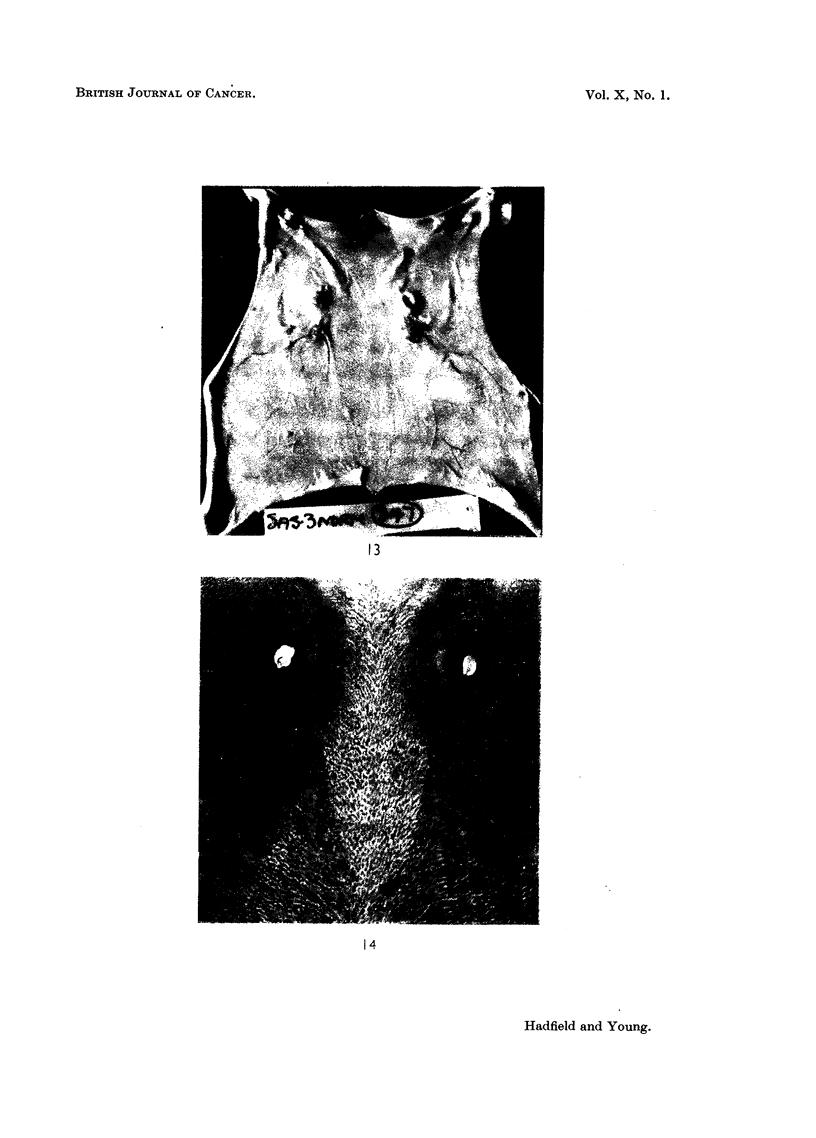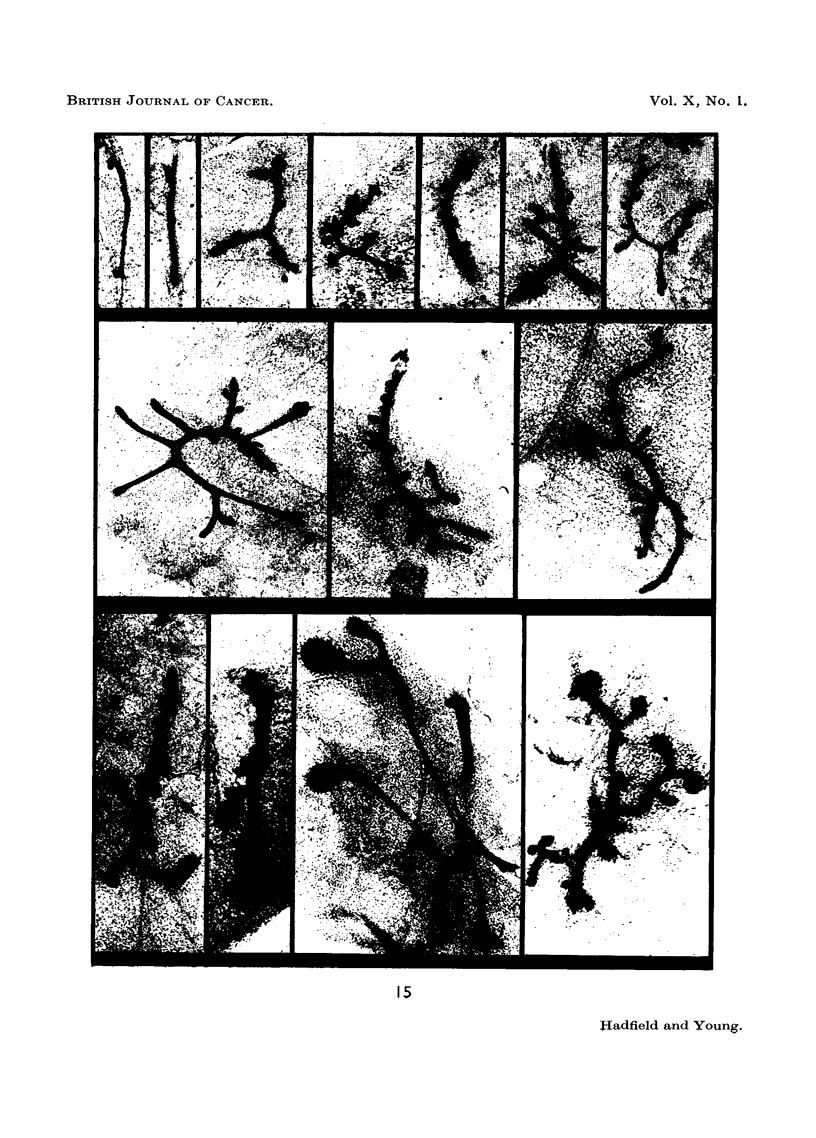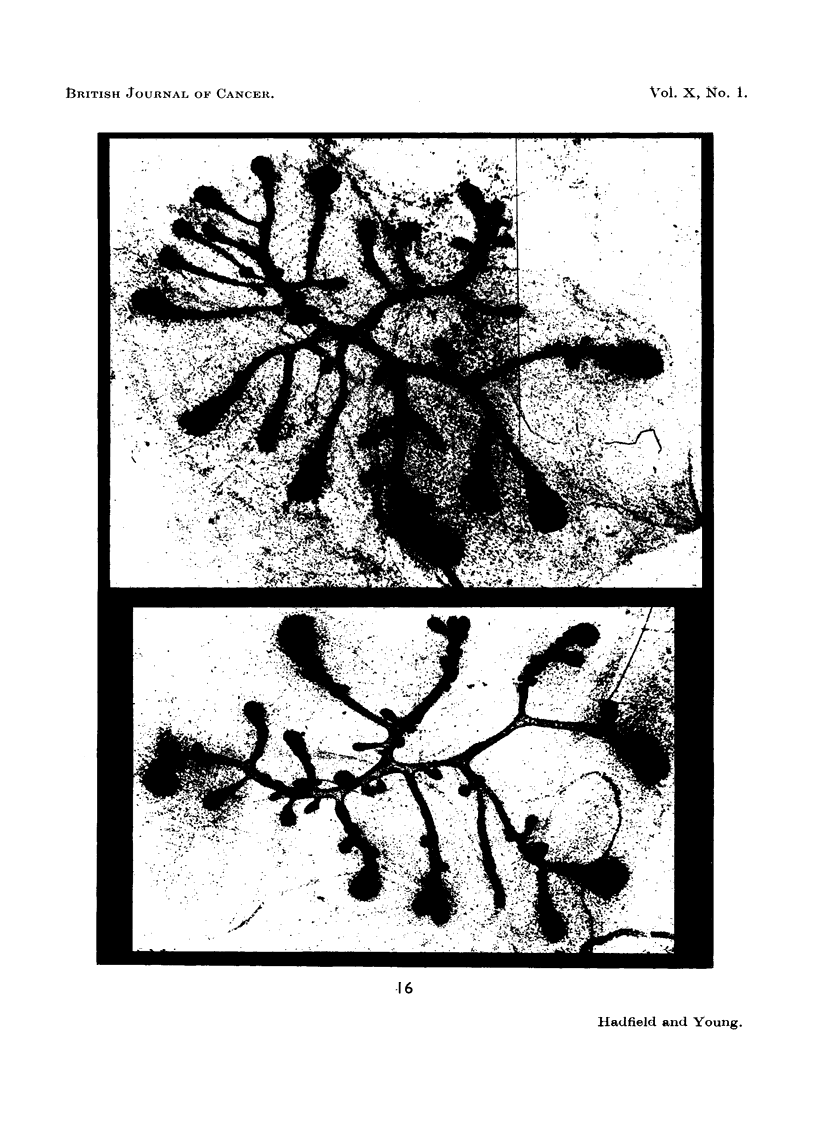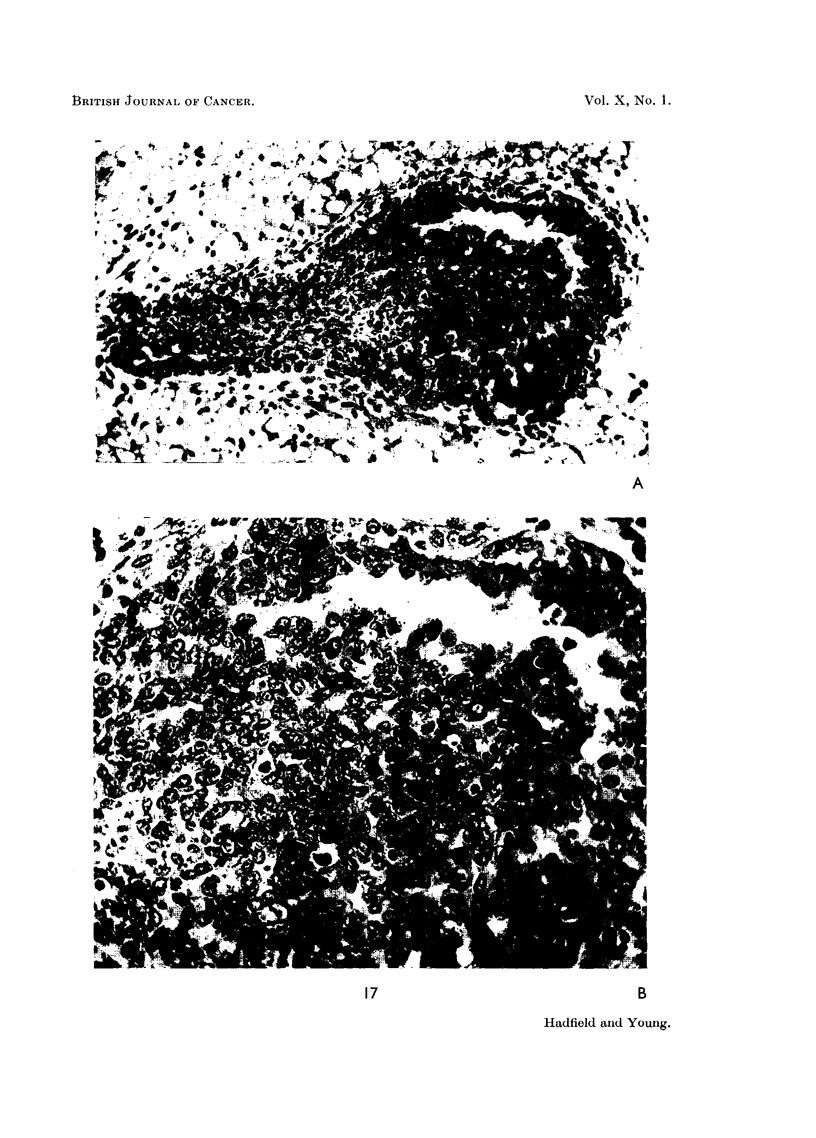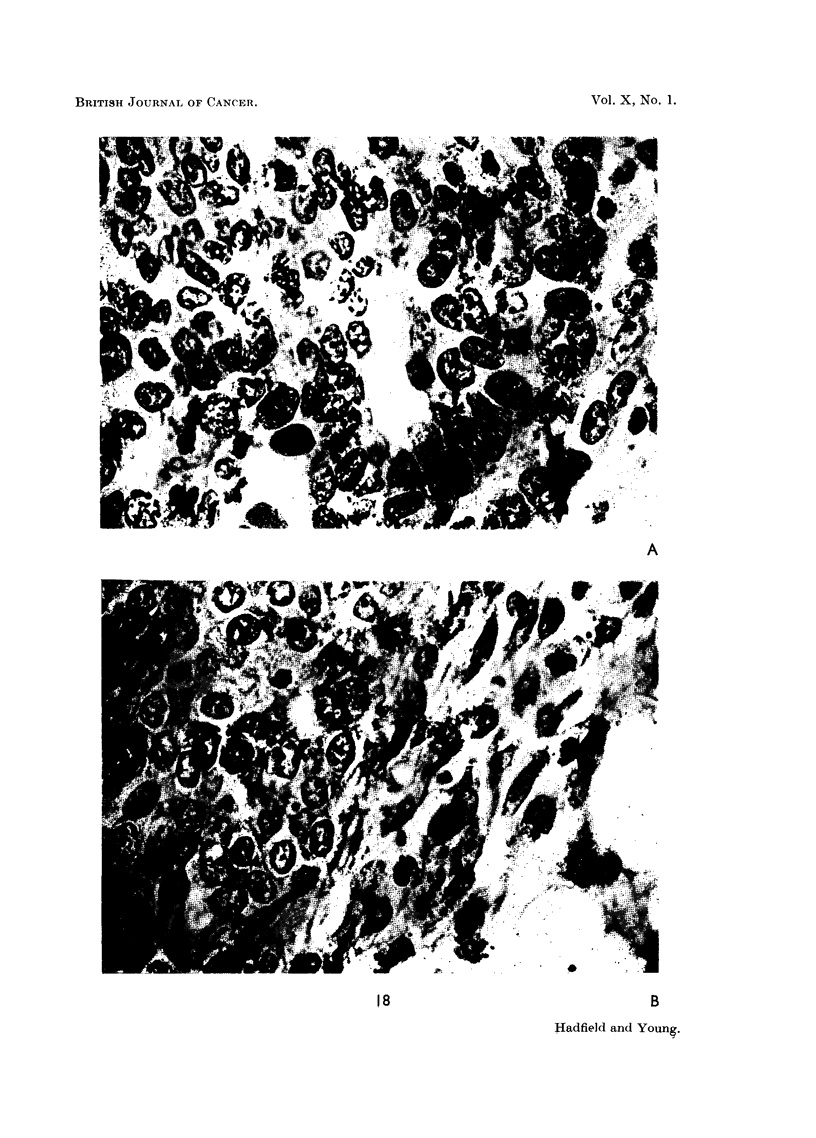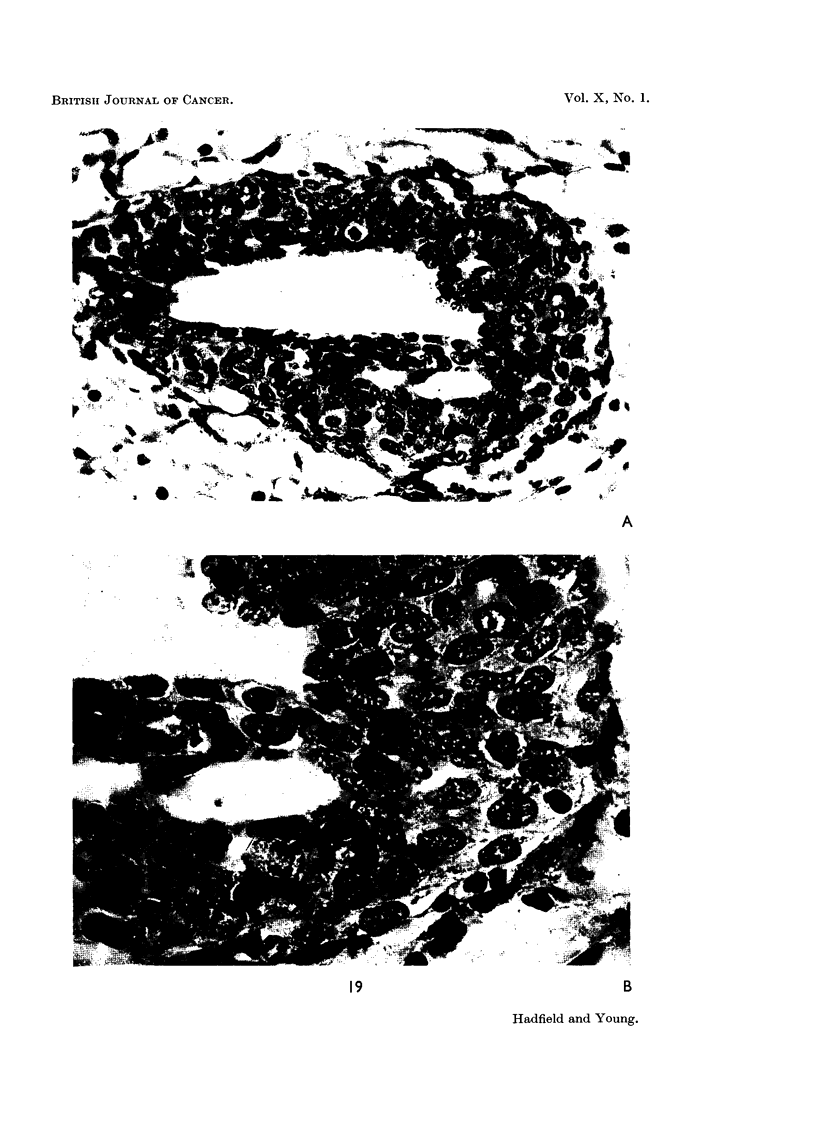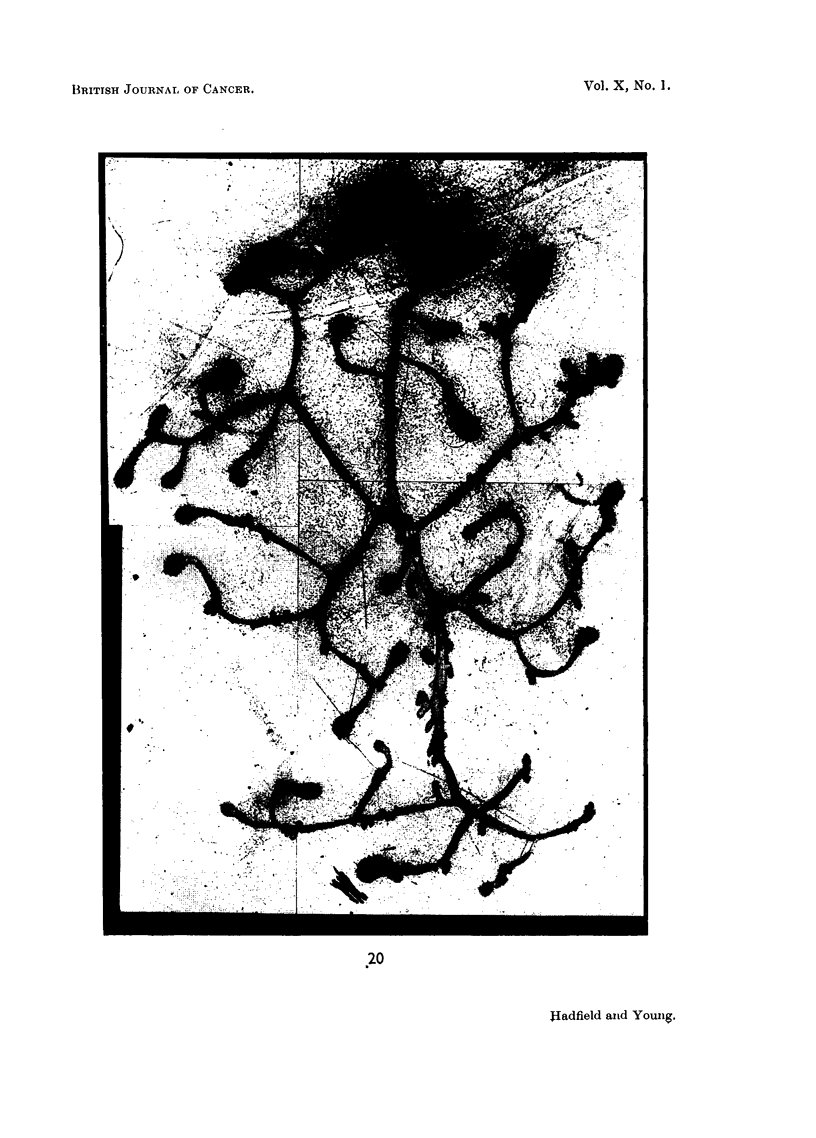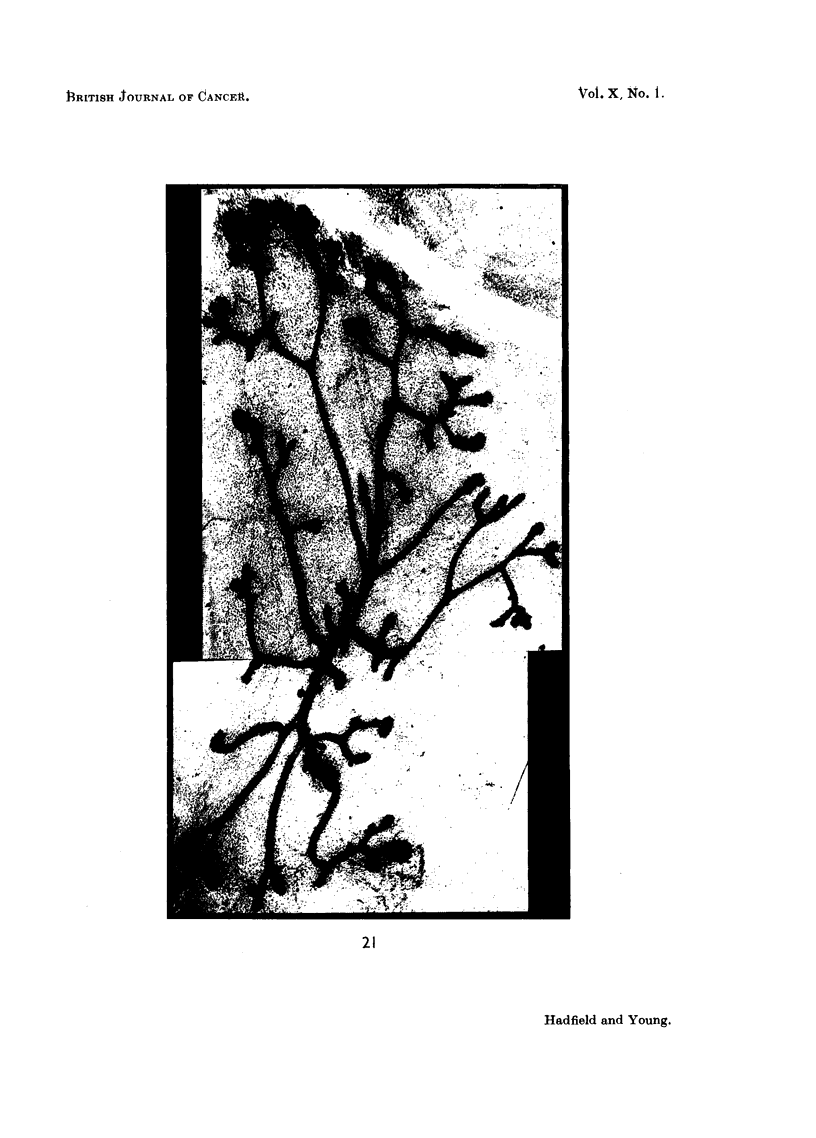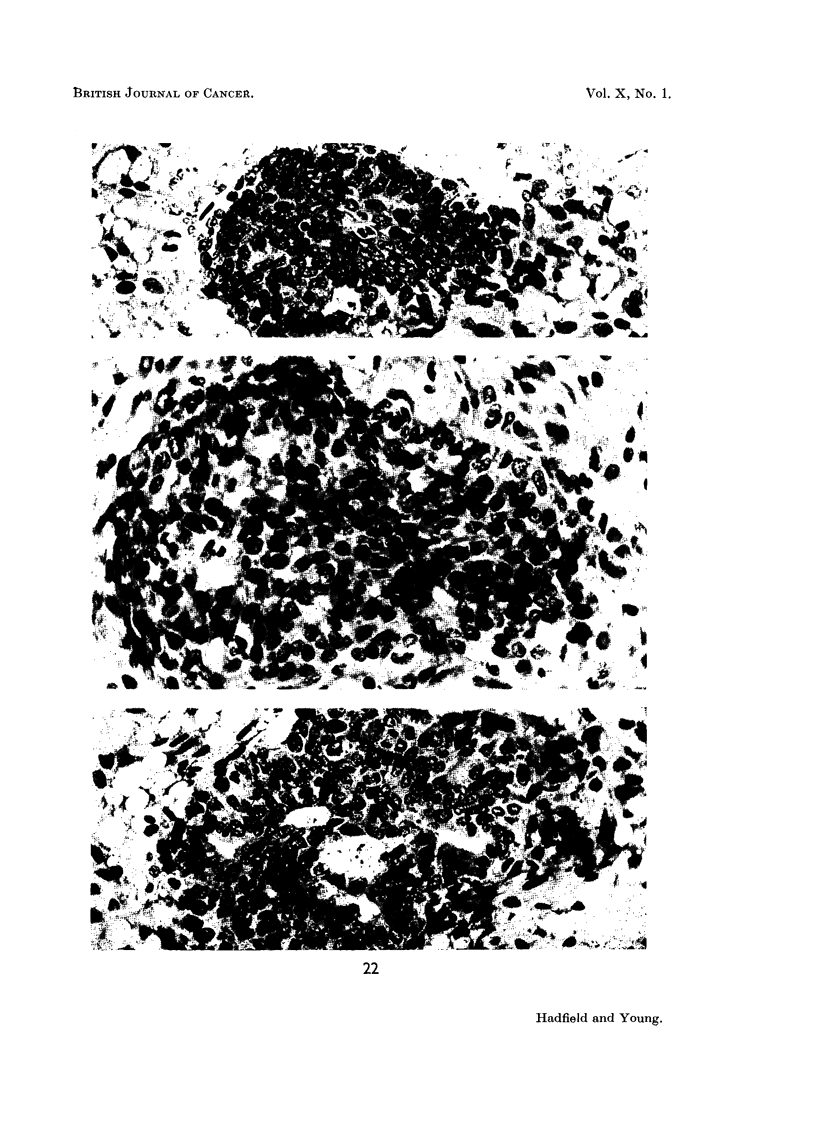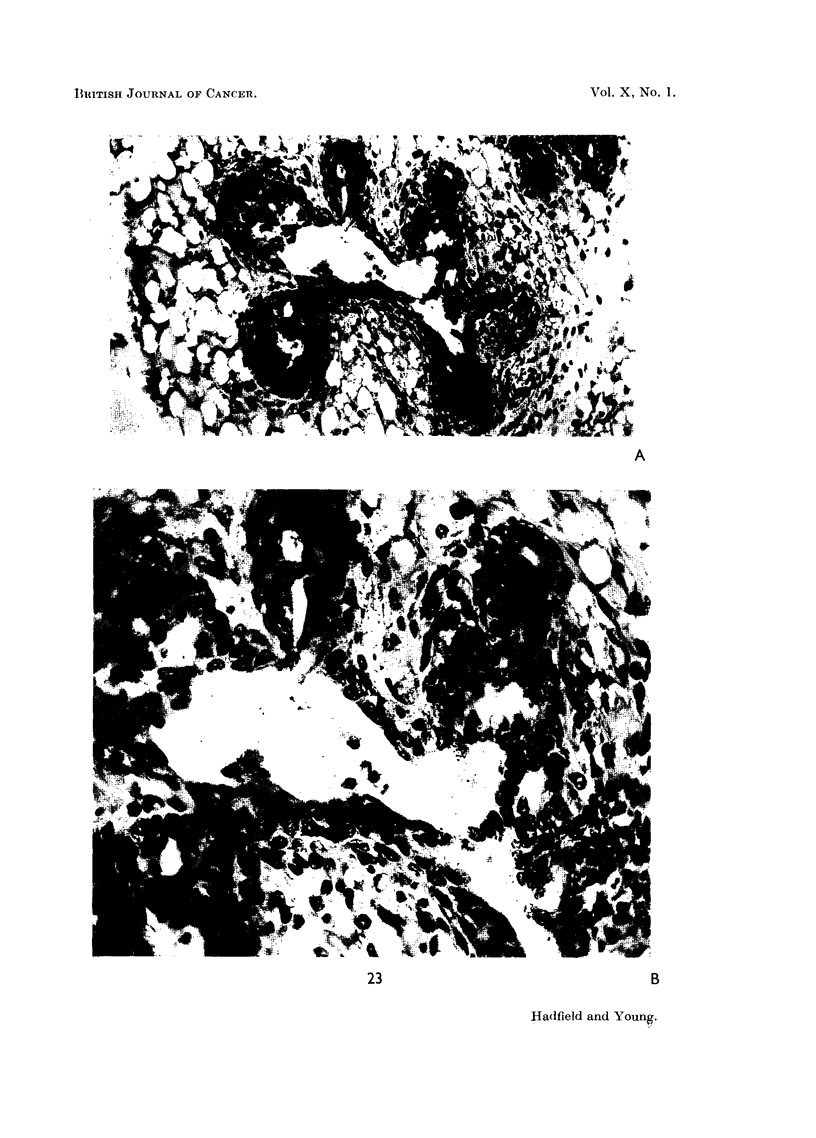# The Mammotrophic Potency of Human Urine

**DOI:** 10.1038/bjc.1956.19

**Published:** 1956-03

**Authors:** Geoffrey Hadfield, J. Stretton Young

## Abstract

**Images:**


### THE MAMMOTROPHIC POTENCY OF HUMAN URINE

GEOFFREY HADFIELD AND J. STRETTON YOUNG

*From the Clinico-pathological Laboratories, Imperial Cancer Research Fund, Royal College of Surgeons of England, London, W.C.2*

Received for publication January 31, 1956

THE experiments recorded in this paper were carried out to control and amplify a short series of preliminary observations which suggested that extracts prepared from the urine of normal pre-menopausal women possess considerable mammotrophic activity and are capable of inducing a significant acceleration of cellular multiplication and differentiation in the hormone dependent epithelium of the mammae of weanling male mice (Scowen and Hadfield, 1955).

The preliminary experiments were carried out in two stages :

I. *Preparation of the urinary extract.*

The protein contained in a 72 hours' specimen of urine was precipitated by alcohol, the precipitated protein extracted by distilled water, the solution dialysed and its protein re-precipitated and dried. This technique follows the familiar Klinefelter bioassay for the estimation of pituitary gonadotrophin in human urine.

II. *The biological experiment*

The dried powder was extracted with enough distilled water to administer twice daily, for five days, by subcutaneous injection, 0·1 ml. of clear supernatant to each of twelve weanling male mice aged between 24 and 25 days.

Each weanling yielded an average of five mammary glands and each of the 58 to 62 glands was examined for the structural changes associated with recent acceleration of growth. Two groups, one of intact normals, the other of oestrone-treated normals, were included in each experiment.

The experiments which form the subject of this present communication have confirmed the original observation and show that normal pre-menopausal female urine possesses considerable mammotrophic potency. The effect of urinary extracts on the growth and development of the weanling male mouse breast has been compared with the changes produced over a period of five days by the ovarian and pituitary hormones known to be concerned with normal mammogenesis. A greatly simplified modification of the original Klinefelter extraction process, which saves time and considerably increases mammotrophic potency, has been introduced. The original technique used for the isolation of the mammary gland has been simplified, and a workable and reasonably accurate method for estimating the degree of recent growth acceleration in the stimulated mammary gland evolved. The output of the mammotrophic agent has been shown to wax and wane during the menstrual cycle.

Klinefelter extracts of human urine contain an uncertain proportion of the total amount of excreted pituitary gonadotrophin. It is reasonable to suppose that they may also contain a mammotrophic protein hormone of pituitary origin whose estimation may throw some light on hypophyseal function. It was largely on the basis of this supposition that the original experiments have been repeated and, having satisfied ourselves that human urine does contain a mammotrophic substance in considerable quantity, we have concentrated much of our efforts in making its estimation adaptable for use in any well-equipped laboratory as part of a complete pathological investigation of patients suffering from breast cancer. In this connection the functional quiescence and rudimentary structure, together with the remarkable reactivity of the mammae of the weanling male, have smoothed out many of the complexities associated with the use of the female mouse at any age and of the adolescent or mature male.

We would like to emphasize that this communication is an account of work in progress, especially with regard to the estimation of mammotrophic activity in a significant number of specimens of urine of all ages and in both sexes.

### THE BIOLOGICAL EXPERIMENT

### *Some General Considerations*

Extracts of human urine tend to have a serious toxic effect when injected into mice and for this reason we have deliberately used a robust commercial breed of albino mice—the Swiss Schneider, obtainable from Messrs. Scientific Animal Supplies, Home Farm, Aldenham Park, Elstree, Hertfordshire—in which the weanlings are strong and well-grown. This choice has, of course, entailed a careful examination of a large number of weanlings of this strain to estimate the degree of normal structural variation in the mammary gland. The details of these control experiments are recorded later in the paper, but it was found in an examination of 1000 normal mammary glands from male weanlings of the Swiss Schneider strain that 78·8 per cent of them were so small and had so rudimentary a structure that a recent, experimentally-produced acceleration of growth could be readily observed and numerically assessed with a satisfactory degree of accuracy. These mammae consist of little more than a slender main duct of varying length and often having a limited number of short primary branches. It is highly characteristic of these glands that they show no " clubs " or end-bulbs at the free ends of their ducts and a relatively small number of duct buds along their length (Fig. 15), and it may be safely assumed that, at this age, they are not and never have been subjected to any significant degree to a specific mammotrophic stimulus. On the other hand, they react vigorously and rapidly to the administration of the oestrogenic ovarian hormones and even more vigorously to large doses of prolactin and to potent extracts of premenopausal urine.

Critical examination of the reacting mouse mamma is greatly facilitated by the fact that all the branches of the reacting duct system lie in almost the same plane with remarkably little overlapping. On the other hand, a long period of stimulation will produce a " feminised " gland of such complexity that a numerical assessment of growth becomes time consuming and difficult. We have found that hormones and urinary extracts produce a satisfactory and easily assessed response after a period of five days giving two injections a day.

### *Isolation and Examination of the Mammary Glands*

The animals are killed by coal gas during the morning after the last evening injection. The skin is removed by making an incision as accurately as possible in the mid-line of the back from the vertex of the skull to the root of the tail. The four paws are cut through and the limbs removed by cutting through just below the elbow and knee joints. In removing the skin the carcase is held firmly, the skin lightly, and the carcase pulled away from the skin. Heavy pressure on the skin may damage the glands, especially those near the axilla in relation with the fore-limb. Each skin is labelled carefully and pinned down, deep surface uppermost, upon a paraffin wax block made by pouring molten paraffin wax to a depth of $\frac{3}{4}$ inch into an enamelled iron meat dish and allowing it to set. A conveniently-sized dish, which will hold a total of 20 skins, measures $17\frac{1}{2} \times 13\frac{1}{2}$ inches and 2 inches in depth. In pinning out, the cut edges of the skin must not be allowed to roll up. Bouin's fluid is then poured into the dish to a depth of 1 inch and the container is covered by a glass plate or another dish. Fixation is complete in 24 hours. The skins are then removed and immersed in 50 per cent alcohol in which they are left overnight and in which they may be stored indefinitely.

FIG. 1.—Diagram showing position of mammary glands. Glands enclosed in rectangle. × 2.

It is the common practice in examining the mammae of mice to separate the gland-bearing fat, aponeurosis and muscle in one sheet from the hair-bearing skin by blunt dissection. This is a simple matter in adults but difficult and time-consuming in weanlings, and frequently results in loss and damage to glands. We therefore search for the mammae in the unstripped skin. As they are small and have no nipples, and their colour is almost the same as the fat in which they lie, their unaided identification in weanlings is tedious and difficult. We therefore stain the whole specimen, including the overlying hair-bearing skin, in alum carmine, which stains the glands deeply, can penetrate the breast fat, and is far preferable to haematoxylin. Each specimen is then examined in 50 per cent alcohol in a Petri dish of convenient size to fit the stage of a Greenough binocular microscope using a magnification of 15 diameters, a green filter and transmitted light from an intense light source. Every effort is made to isolate six glands from each animal. Fig. 1, 13, 14, (diagram and colour plates) show the position and direction of the " milk line " in relation to the anterior mid-line, the " arm hole " of the forepaw, the fan-shaped muscle which becomes prominent after treatment with 50 per cent alcohol, and the prominent vascular markings which are best seen in the unfixed skin. As each stained gland is identified it is cleaned of fat and muscle and cut out of the pelt, leaving a liberal margin of tissue to facilitate handling with forceps. The glands are dehydrated, cleared and mounted whole and unsectioned, preferably in the same relative positions on the slide which they occupied in the animal. One gland, usually the first thoracic, is occasionally congenitally absent.

### *Estimation of Recent Growth Acceleration*

In the strain we use the weanling male mammae remain rudimentary from weaning at the 21st day to about the 34th day; they then grow slowly up to sexual maturity at about 90 days. During this period of approximately 56 days a fairly intricate duct system develops, the gland acquiring the same structural complexity as that of the female weanling. It is clear, therefore, that the mammae of this strain are being subjected to a continuous mild mammotrophic stimulus during adolescence and this raises two important considerations.

In the first place, as the male adolescent mammae slowly develop they become increasingly insensitive to the injection of hormones as the gonads and adrenal cortex come into full activity. In any case, the adolescent male mammae in the strain we used was almost invariably so complex in structure that any estimation of recent growth acceleration was difficult, time-consuming and probably fallacious.

Secondly, the penetration of the breast fat by the growing duct system in the adolescent male is achieved without the formation of terminal clubs. This is in the sharpest possible contrast with the mode of growth observed when the highly reactive weanling gland is appropriately and powerfully stimulated. In a few days a duct system is then produced all of whose numerous branches terminate in a deeply-stained and highly cellular club or even a closely-set cluster of gland alveoli. It is clear, therefore, that the free and rapid formation of terminal clubs is a certain indication of recent and rapid mammary growth, and in glands which before stimulation were " clubless ", a simple enumeration of recently formed clubs should give an accurate measure of recent growth acceleration produced by an experimental stimulus.

The situation is, however, complicated by the fact that in all strains of albino mice a low but variable percentage of the mammae of the weanling males have a growing duct system with secondary and even tertiary branches, a considerable number of which bear clubs, whilst duct buds are visible in their lateral walls (Fig. 15). In 1000 mammary glands from male weanlings of the strain we used 21·3 per cent were bearing clubs. Their distribution is shown in Table I. In a total of 1000 glands, the average number of clubs per gland was 1·14 and it is highly significant that *in no animal was there any evidence of differentiation into glandular acini or lobules.*

Oestrone and prolactin over a wide range of dosage, as well as potent extracts prepared from human urine, will increase this average number of clubs per gland from five to twentyfold; when this figure is high it is always possible to see clear signs of differentiation into glandular alveoli even when the gland is mounted whole without sectioning and examined under low magnification.

It is clear, therefore, that if an adequate number of mice is used for each experiment the period of injection limited to five days, and a correction made for the " freak " mice who normally have club-bearing glands, an estimate of the average number of clubs in an adequate number of glands should provide a measure of the degree of recent growth acceleration produced by the injection of a mammotrophic hormone.

Strong support for this conclusion is provided by histological examination. Club formation is a process of intense cellular activity involving the epithelial and mesenchymatous elements of the whole duct system during a period of rapid growth. During the growth phase the club consists of an almost solid mass of rapidly proliferating undifferentiated epithelial cells in which mitoses are frequent, nuclear hyperchromatism obvious and multipolar mitoses not uncommon (Fig. 17). There is a similar but less spectacular proliferation of the epithelium and mesenchyme of the whole duct system, the former, for some distance behind each rapidly enlarging club, becoming four to five cells thick. The club becomes invested by a thick, ill-defined collar of amorphous ground substance in which undifferentiated proliferating mesenchymal cells and many immature fibroblasts are embedded (Fig. 18). This amorphous collar rapidly becomes finely fibrillated and the reaction again involves the whole duct system, whilst the breast fat becomes rapidly transformed into cellular and finely fibrillated young connective tissue. This change is intense in the region of the clubs and of the newly-formed duct buds, the large majority of which in a recently reacting gland acquire a deeply-staining terminal club as soon as they erupt through the lateral walls of the ducts. The almost sessile club then penetrates the breast fat, spinning out duct epithelium behind it, and it is typical that during the growth phase the elongating branches have a clear, well-defined outer boundary.

If a powerful mammotrophic stimulus is maintained for five days the rapid production of an adequate duct system by the prolific formation of large cellular clubs comes to an end, probably about the 3rd day. Growth then gives way to differentiation. When prolactin and urinary extracts are used the growth phase begins to abate when the greatly enlarged gland is carrying a total of between 10 and 30 clubs. Differentiation is not a uniform process. It is often delayed in the peripheral part of the gland. The branches cease growing in length and width, lose their sharply defined external outline, and a series of small, closely-set, irregular masses project from their lateral walls. Each small mass is a group of glandular acini. When this appearance has developed, the terminal club of an affected branch shrinks, becomes irregular in shape and is transformed into a primitive terminal glandular lobule (Fig. 2). Under the influence of prolactin at an optimum dose level, and of potent urinary extracts, up to 60 per cent of the branches of a powerfully stimulated gland show this process of glandular differentiation after a period of five days' stimulation.

The enumeration of clubs in a rapidly growing gland presents a few difficulties. A large terminal club is unmistakable, but it not infrequently happens that such a club divides into two and just before separation the club is "bifid". A bifid form has been counted as two clubs.

It is sometimes difficult to distinguish between a developing duct bud and a very recently formed and almost sessile club. The bud first appears as a prominent, hemispherical swelling in the duct wall. It will rapidly become either a short unclubbed branch or a young club with a very short stalk. In the first case its free end will be bluntly pointed, it will stain uniformly and its walls will be parallel. Very short branches may develop clubs as soon as the duct bud erupts. The "stalk" may be difficult to see, but the club is almost spherical and deeply stained whilst the stalk stains less deeply.

FIG. 2.—Diagrams of duct systems. × 20.

The ratio $\frac{\text{total clubs}}{\text{total glands}}$ in a batch of similarly treated animals gives the average number of clubs per gland. The corrected ratio $\frac{\text{total clubs}}{\text{total glands} \times 1{\cdot}14}$ (1·14 being the average number of clubs per normal gland) would then be proportional to a recent acceleration of mammary growth in a group of injected animals and serve as a measure of the potency of a mammotrophic agent. This figure is referred to in the tabulated reports of experiments as " Mammotrophic Potency ".

Glands in which the development of glandular acini has started present a peculiar difficulty. Differentiation leads to regression in size of all clubs and a reduction in width of their branches in that part of the gland in which differentiation is proceeding. The diminishing club, however, retains its avidity for nuclear stains, but it may lose much of the clear regular outline it possesses during the growth phase. When glandular alveoli develop at the end of a duct they can only do so by replacing a club. We have therefore counted a cluster of terminal acini as a club. Glandular differentiation has been expressed in our experiments by estimating the percentage of glands in which alveolar formation is present and in such glands by estimating the approximate percentage of branches in which this was found.

Dr. J. W. Trevan has analysed our data relating to these normal animals and reports as follows :

> " An approximate statistical analysis has been made of the structural variation in the mammae of normal weanlings. If groups of 10 mice are used, the average number of clubs per normal gland for each such group will be less than 8 and probably not greater than 4 in 98 per cent of such groups. A more detailed analysis is in course of preparation."

We have therefore regarded a " mammotrophic potency " of 4 and over as being significant.

The following gives a summary of the more important general details of the biological experiment :

| | |
|---|---|
| Age of mice when received | 21 days |
| Injections started on | 24th to 25th day |
| Average weight at | |
| Commencement of experiment | 9·0 to 10·5 g. |
| End of experiment | 10·0 to 14·0 g. |
| Number of animals per test | 10 |
| Volume of injection | 0·1 to 0·2 ml. |
| Injection period | 5 days |
| Number of injections | 2 per day : total, 10 |
| Fixation period (Bouin's fluid) | 24 hours. |
| Whole pelts stained in Grenacher's Alum carmine. | |
| Expected yield of glands | 48 to 52. |
| Average clubs per gland | $\frac{\text{Total clubs}}{\text{Total glands}}$ |
| Mammotrophic potency | $\frac{\text{Average clubs per gland}}{1{\cdot}14^{*}}$ |

* 1·14 = the average number of clubs per normal gland.

### VARIATION IN STRUCTURAL COMPLEXITY OF THE MAMMARY GLANDS OF THE NORMAL MALE WEANLING

A thousand mammary glands from 205 mice were examined. The animals were in ten groups and for the most part had served as the normal controls of a series of experiments in which either a hormone or a urinary extract was injected. These experiments extended over a period of six months; the results are summarised in Table I.

TABLE I.

| | |
|---|---|
| Total mice | 205 |
| Total glands | 1000 |
| Average glands per mouse | 4·9 |
| Number of unclubbed glands | 787 = 78·7 per cent of all glands |
| Number of club-bearing glands | 213 = 21·3 per cent of all glands |
| Average number of clubs per gland (all glands) | 1·136 |

Distribution of clubs:

| Number of clubs. | Number of glands. | Percentage of all glands. | Percentage of clubbed glands. |
|---|---|---|---|
| 1 to 5 | 128 | 12·8 | 60 |
| 6 to 10 | 59 | 5·9 | 28 |
| 11 to 15 | 18 | 1·8 | 8 |
| 16 to 20 | 8 | 0·8 | 4 |

Maximum number of clubs borne by any normal gland = 19, i.e. an incidence of 0·1 per cent of all glands.

Fig. 3 is a histogram showing the distribution of glands in this control series.

*Total clubs per mouse.*

The clubs were distributed among 205 normal mice in two ways. When the total number of clubs in all the glands of any one animal was between 1 and 8, two or more glands from this animal were often unclubbed. As the number of clubs per mouse increased they showed a rapidly increasing tendency to be distributed amongst all the glands of a relatively small number of "freak" animals.

TABLE II.

| Total clubs: All glands per mouse. | Number of mice in each group. | Percentage of all mice. |
|---|---|---|
| 0 | 123 | 60·0 |
| 1– 5 | 38 | 18·5 |
| 6–10 | 12 | 6·0 |
| 11–15 | 9 | 4·3 |
| 16–20 | 2 | 1·0 |
| 21–25 | 8 | 4·0 |
| 26–30 | 2 | 1·0 |
| 31–35 | — | — |
| 36–40 | 3 | 1·5 |
| 41–45 | 2 | 1·0 |
| 46–50 | 2 | 1·0 |
| 51–55 | 2 | 1·0 |
| 56–60 | 1 | 0·5 |
| 61–65 | — | — |
| 66–70 | — | — |
| 71–75 | — | — |
| 76–80 | 1 | 0·5 |

This is largely responsible for the wide variation in the club counts obtained in any group of mammary glands reacting to a potent mammotrophic stimulus. The variation in the distribution of clubs per mouse is shown in Table II.

FIG. 3.

FIG. 4.

FIG. 3.—Histogram showing normal variation in glandular complexity. 0 = glands having no clubs ; A = 1 to 5 clubs ; B = 6 to 10 clubs ; C = 11 to 15 ; D = 16 to 20.

FIG. 4.—Tracings of low magnification photomicrographs of two mammary glands from an animal given a total dose of 160 $\mu$g. oestrone over five days. Both glands were of the most primitive form before stimulation ; the growth phase appears to have been inhibited and premature acinisation has succeeded it. $\times$ 50.

Table III records the club count of the mammae of 15 weanlings selected at random from our normal control series. It will be seen that the large majority of clubs found in the 86 glands are derived from two " freak " animals, No. 133 and 134.

TABLE III.

| Mouse No. | Total glands per mouse. | Clubs per gland. 1 | 2 | 3 | 4 | 5 | 6 | Total clubs per mouse. |
|---|---|---|---|---|---|---|---|---|
| 126 | 5 | 0 | 0 | 0 | — | 0 | 0 | 0 |
| 127 | 6 | 0 | 0 | 0 | 0 | 0 | 0 | 0 |
| 128 | 6 | 0 | 0 | 0 | 0 | 0 | 0 | 0 |
| 129 | 6 | 2 | 0 | 4 | 0 | 1 | 1 | 8 |
| 130 | 6 | 0 | 0 | 0 | 0 | 0 | 0 | 0 |
| 131 | 6 | 0 | 0 | 0 | 0 | 0 | 0 | 0 |
| 132 | 5 | 0 | — | 0 | 0 | 0 | 0 | 0 |
| 133 | 6 | 9 | 9 | 11 | 0 | 10 | 9 | 48 |
| 134 | 6 | 7 | 5 | 8 | 6 | 11 | 8 | 45 |
| 135 | 5 | 0 | — | 0 | 0 | 0 | 0 | 0 |
| 136 | 6 | 0 | 0 | 0 | 0 | 0 | 0 | 0 |
| 137 | 6 | 0 | 0 | 0 | 0 | 0 | 0 | 0 |
| 138 | 6 | 0 | 0 | 0 | 0 | 0 | 0 | 0 |
| 139 | 6 | 2 | 0 | 1 | 0 | 0 | 0 | 3 |
| 140 | 5 | 0 | 0 | 0 | 0 | 0 | 0 | 0 |

*Variation in response*

When a group of about 50 reacting mammae is critically examined they present a confusing variety in general configuration and wide variations in intensity of the growth response. The first feature is due to the fact that growing glands normally develop according to a series of constant structural patterns which are determined purely by their anatomical situation. The number of branches, duct buds, clubs and glandular acini produced by a stimulated gland, whatever its anatomical pattern may be, is determined by its normal structural complexity before stimulation. Glands which consist of a minute, slender unbranched main duct lag behind others which, although not clubbed, have a thickened main duct, several branches and duct buds. The following protocol of an experiment demonstrates the mammotrophic potency of a urinary extract and illustrates the typical wide variation in response of 51 mammary glands from 10 weanling males. The reaction was judged by enumerating the clubs in each gland and estimating the average number of clubs per gland.

*Experiment No.* 131

*Urinary extract.—Normal pre-menopausal woman. Days* 13, 14 *and* 15 *of cycle.* 0·15 *ml. injected twice daily for* 5 *days.*

| Mouse No. | Number of glands. | Club count. 1 | 2 | 3 | 4 | 5 | 6 | Total. |
|---|---|---|---|---|---|---|---|---|
| 1 | 4 | 12 | 0 | 0 | 11 | — | — | 23 |
| 2 | 6 | 0 | 0 | 0 | 0 | 0 | 0 | 0 |
| 3 | 5 | 18 | 16 | 23 | 14 | 14 | — | 85 |
| 4 | 6 | 18 | 24 | 25 | 42 | 20 | 26 | 155 |
| 5 | 6 | 21 | 37 | 8 | 20 | 31 | 22 | 139 |
| 6 | 5 | 29 | 24 | 3 | 7 | 17 | — | 80 |
| 7 | 5 | 0 | 3 | 0 | 0 | 3 | — | 6 |
| 8 | 4 | 26 | 21 | 14 | 18 | — | — | 79 |
| 9 | 4 | 35 | 15 | 24 | 35 | — | — | 109 |
| 10 | 6 | 22 | 14 | 14 | 22 | 16 | 3 | 91 |
| | 51 | | | | | | | 767 |

T.C./T.G. = 767/51 = 15·0 average clubs per gland. Mammotrophic potency = 13·15.

RESPONSE OF THE MALE WEANLING MAMMA TO OESTRONE

Oestrone dissolved in propylene glycol was injected twice daily for five days over a total dose range from 0·005 $\mu$g. to 160 $\mu$g.—i.e. from 0·001 $\mu$g. to 32 $\mu$g. per day.

Satisfactory responses were obtained until the highest doses were approached; these produced a significant degree of inhibition of the growth phase. In reviewing the whole series it was obvious that the degree of glandular differentiation into alveoli and lobules produced by oestrone in five days was significantly less than that produced by prolactin. Another series of experiments was carried out, extending the period of administration of oestrone from five to twenty days and killing a batch of animals every two days. At about the 8th day glandular differentiation was established and by the 14th day the mammary glands were almost fully "feminised". This period of inhibition may be significant as the injection of urinary extracts over a five days' period frequently produces the same widespread formation of glandular acini and lobules as prolactin. It will be recalled that "acinisation" is never seen in normal glands in the strain we used.

There was no significant difference between the capacity of oestrone, of prolactin and urinary extracts to stimulate cellular multiplication with the production of an adequate duct system. There were, however, significant differences in the ability of these three agents to induce "rapid" glandular differentiation over a period as short as five days.

The optimum response in growth and differentiation produced by oestrone was obtained at a total dose range of 0·20 $\mu$g. over five days, i.e. 0·04 $\mu$g. per day. The detailed protocol of this response is shown in Experiment 78.

*Experiment No.* 78

*Oestrone in propylene glycol.—Total dose* 0·2 $\mu$*g. over* 5 *days.*

| Mouse No. | Number of glands. | Club count. 1 | 2 | 3 | 4 | 5 | 6 | Total. |
|---|---|---|---|---|---|---|---|---|
| 1 | 5 | 11 | 15 | 16 | 20 | 15 | — | 77 |
| 2 | 6 | 4 | 10 | 13 | 14 | 8 | 15 | 64 |
| 3 | 6 | 28 | 11 | 27 | 20 | 20 | 19 | 125 |
| 4 | 6 | 6 | 16 | 12 | 12 | 13 | 5 | 64 |
| 5 | 5 | 28 | 8 | 18 | 23 | 37 | — | 114 |
| 6 | 5 | 7 | 4 | 9 | 12 | 19 | — | 51 |
| 7 | 5 | 12 | 17 | 15 | 15 | 21 | — | 80 |
| 8 | 6 | 23 | 30 | 32 | 23 | 20 | 28 | 156 |
| 9 | 6 | 7 | 24 | 31 | 31 | 18 | 8 | 119 |
| 10 | 5 | 11 | 13 | 24 | 18 | 11 | — | 77 |
| | 55 | | | | | | | 927 |

T.C./T.G. = 927/55. Mammotrophic potency = 14·7. No glands remained unclubbed. 23·6 per cent of all glands show alveolar formation.

As oestrone dissolved in propylene glycol was used in all our oestrogen control experiments, its absorption was rapid and our results are not therefore comparable with experiments in which the solvent was oily. The salient features of the oestrone response at all dose levels are shown in Table IV.

TABLE IV.—*Oestrone Response at all Dose Levels*

| Experiment number. | Total dose : μg. for 5 days. | Total glands. | Total clubs. | Mammotrophic potency. | Percentage glands showing acinar formation. | Percentage "unclubbed" glands. |
|---|---|---|---|---|---|---|
| 76 | 0·005 | 48 | 238 | 4·3 | Nil | 20·8 |
| 22 | 0·023 | 22 | 238 | 9·14 | 5·0 | Nil |
| 77 | 0·05 | 48 | 554 | 10·0 | 4·7 | 4·2 |
| 78 | 0·2 | 55 | 927 | 14·7 | 23·6 | 5·4 |
| 79 | 0·8 | 38 | 578 | 13·3 | 13·2 | 2·6 |
| 24 | 2·23 | 22 | 331 | 13·1 | 8·0 | Nil |
| 80 | 3·2 | 56 | 931 | 14·56 | 10·0 | Nil |
| 25 | 10·53 | 22 | 361 | 14·53 | 13·8 | Nil |
| 26 | 31·35 | 25 | 396 | 13·85 | 4·2 | Nil |
| 27 | 62·7 | 21 | 243 | 10·1 | 3·0 | Nil |
| 159, 160 | 160·0 | 82 | 538 | 5·7 | 62·2 | 13·7 |

The distribution of clubs in batches of weanlings given oestrone at varying dose levels is recorded in the histograms (Fig. 5 and 6).

FIG. 5.

FIG. 6.

FIG. 5, 6.—Histograms showing distribution of clubs in oestrone treated glands. 0 = no clubs ; A = 1–5 clubs ; B = 6–10 clubs ; C = 11–15 clubs ; D = 16–20 clubs ; E = 21–25 clubs ; F = 26–30 clubs ; G = 31–35 clubs ; H = 36–40 clubs. Fig. 5 : Dose =0·2 μg. total over 5 days period. Fig. 6 : Dose = 3·2 μg. total over 5 days period.

*Premature acinisation*

The morphological reactions in the mammae of oestrone-treated weanlings show significant differences as the dose increases. From a total dose of 0·005 μg. over a period of five days to one of 31·35 μg. for that period, the glands cover a large area, the branches are long, the clubs are large and deeply staining, and the general picture is one of rapid growth rather than differentiation (Fig. 16). The histological appearances of the clubs and the reacting stroma bear this out (Fig. 17 and 18). Glandular differentiation, however, becomes increasingly obvious as the dose rises but is never so marked at this level as with prolactin, and is almost invariably seen in large, heavily clubbed glands which are nearing the completion of the growth phase and have produced an adequate duct system. At a dose of 62·7 μg. over a period of five days, a significant morphological picture begins to develop. The production of large, deeply-stained clubs is still the predominant process but the branches are shorter and the glands as a whole cover a much smaller area. The general appearances strongly suggest an arrest of the development of an adequate duct system and in this respect many glands are obviously immature. Some of these partly developed glands show extensive and unmistakable glandular differentiation. We have called this remarkable appearance *premature acinisation.* It becomes a prominent feature of the response to total doses of 160 μg. for five days (Fig. 4). At this dose level an appreciable number of glands is seen whose area is little more than that of a rudimentary unstimulated gland. Such glands carry only a few recognisable clubs, others have been replaced by a cluster of terminal acini and acinar formation along the length of the duct is well developed. This phenomenon clearly accounts for the decided fall in the number of clubs per gland at the two higher levels of dosage.

As we used oestrone dissolved in propylene glycol, it was impossible to exceed a total dose of 160 μg. over five days, as at that concentration the limit of solubility is very close. It seems probable, however, that comparatively large doses of oestrone would further increase this inhibition of the growth phase and elicit the phenomenon of premature acinisation on a wider scale.

We regard this phenomenon as significant in relation to the general problem of mammogenesis for glandular differentiation, both nature and pre-mature, is characteristic of the response not only to prolactin but also to that produced by urinary extracts and unextracted urine, and we would emphasize that oestrone alone given over a five days' period never produces the same degree of glandular differentiation as prolactin and urinary extracts, but will do so after a period of eight to ten days. It is tempting to speculate that the action of oestrone on the mamma is primarily mitogenic, that differentiation is brought about by prolactin, and that oestrone is directly or indirectly responsible for the liberation of prolactin by hypophyseal stimulation.

### RESPONSE OF THE MALE WEANLING MAMMA TO PROLACTIN

The prolactin used in these experiments was provided by Dr. A. T Cowie, by arrangement with Dr. S. J. Folley. It was recently prepared by Dr. T. R. Bradley in the National Institute for Research in Dairying and has an activity of about 10 i.u./mg. by the intraduct injection method in the rabbit. Dr. Bradley thought its most likely contaminants to be ACTH and melanophore hormone. The powdered hormone was dissolved in a few drops of 0·1 N NaOH and diluted by normal saline.

The experiments were carried out at ten dose levels ranging from a total of 0·00078 μg. to 10,000 μg. over five days, i.e. from 0·00015 μg. to 2000 μg. per day. We regard the reaction obtained at the lowest level as significant because 4 per cent of 45 glands showed alveolar formation. The optimal growth response was obtained at a level of 5000 μg. over five days (Fig. 19), the optimal glandular differentiation response was obtained at a dose of 10,000 μg. over five days, (Fig. 20). At the 5000 μg. level the club production compared with normal controls was increased twentyfold. This is the maximum degree of growth acceleration obtained in any of our experiments, but figures closely approaching these, with similar degrees of differentiation and premature acinisation, have been obtained with urinary extracts. At 10,000 μg. 60 per cent of 50 glands showed well developed acinar formation. Premature acinisation was seen at all dose levels above 100 μg.

given over 5 days. Table V shows the essential details of the prolactin control experiments. The histograms shown in Fig. 7, 8, 9 illustrate the distribution of clubs in the reacting glands at various dose levels. The following is a protocol of one experiment in which prolactin was given at a high dose level.

Fig. 7.

Fig. 8.

Fig. 9.

Fig. 7, 8, 9.—Histograms showing distribution of clubs in prolactin treated glands. 0 = no clubs ; A = 1–5 ; B = 6–10 ; C = 11–15 ; D = 16–20 ; E = 21–25 ; F = 26–30 ; G = 31–35 ; H = 36–40 ; J = 41–45 ; K = 46–50 ; L = 51–55 ; M = 56–60 ; N = 61–65. Fig. 7 : 0·05 μg. total dose given over period of 5 days. Fig. 8 : 5,000 μg. total dose given over period of 5 days. Fig. 9 : 10,000 μg. total dose given over period of 5 days.

*Experiment No.* 106

*Prolactin.—Total dose* = 10,000 *μg. over* 5 *days.*

| Mouse No. | Number of glands. | Club count. 1 | 2 | 3 | 4 | 5 | 6 | Total. |
|---|---|---|---|---|---|---|---|---|
| 1 | 5 | 19* | 21* | 15* | 26* | 27* | — | 108 |
| 2 | 4 | 21 | 10 | 9 | 11 | — | — | 51 |
| 3 | 6 | 25* | 23* | 50* | 35* | 35* | 33* | 201 |
| 4 | 6 | 31* | 27* | 21* | 25* | 17* | 44* | 165 |
| 5 | 5 | 17 | 13 | 17 | 12 | 16 | — | 75 |
| 6 | 4 | 26* | 28* | 26* | 31* | — | — | 111 |
| 7 | 5 | 46* | 20* | 10 | 14 | 12 | — | 102 |
| 8 | 5 | 28* | 15 | 32* | 12 | 21 | — | 108 |
| 9 | 5 | 13 | 18* | 6 | 20* | 10 | — | 67 |
| 10 | 5 | 35* | 29* | 33* | 19 | 38* | — | 154 |
| | 50 | | | | | | | 1142 |

T.C./T.G. = 1142/50. Mammotrophic potency = 20·0. No glands remained unclubbed. 60 per cent of all glands show acinar formation (indicated *).

TABLE V.—*Prolactin Response at Various Dose Levels.*

| Experiment number. | Total dose : μg. for 5 days. | Total glands. | Total clubs. | Mammotrophic potency. | Percentage glands showing acinar formation. | Percentage "unclubbed" glands. |
|---|---|---|---|---|---|---|
| 101 | 0·00078 | 45 | 150 | 2·89 | 4·0 | 44·0 |
| 102 | 0·003125 | 46 | 176 | 3·3 | 4·0 | 39·0 |
| 72 | 0·0125 | 52 | 374 | 6·3 | 13·0 | 17·0 |
| 73 | 0·05 | 50 | 483 | 8·47 | 12·0 | Nil |
| 74 | 0·2 | 56 | 811 | 12·7 | 25·0 | ,, |
| 75 | 0·8 | 42 | 657 | 13·7 | 30·0 | ,, |
| 104 | 100·0 | 48 | 732 | 13·3 | 26·0 | ,, |
| 105 | 1000·0 | 52 | 1090 | 18·4 | 46·0 | ,, |
| 49 | 5000·0 | 28 | 668 | 20·87 | 44·0 | ,, |
| 106 | 10000·0 | 50 | 1142 | 20·03 | 60·0 | ,, |

RESPONSE OF THE MALE WEANLING MAMMA TO GROWTH HORMONE

Two preparations were used for this experiment. The first was a standard lyophilised preparation supplied by Messrs. Armour Laboratories (Lot. No. R377174), issued in vials, each containing the equivalent of 50 mg. of the Armour Standard. The preparation is freely soluble in distilled water. Six groups of mice received the following doses over a period of five days, 0·1 ml. of solution being given twice daily. The results are recorded in Table VI.

TABLE VI.

| Experiment number. | Total dose μg. over 5 days. | Mammotrophic potency. |
|---|---|---|
| 16 | 0·1 | 2·89 |
| 17 | 1·0 | 1·38 |
| 18 | 10·0 | 1·55 |
| 19 | 50·0 | 2·0 |
| 20 | 150·0 | 2·45 |
| 21 | 300·0 | 1·53 |

None of these figures can be regarded as significant.

The experiments were therefore repeated using a preparation supplied by Dr. A. L. Greenbaum of University College, London. This preparation was known to be active by weight increase tests. Our results are shown in Table VII.

TABLE VII.

| Experiment Number. | Total dose μg. over 5 days. | Mammotrophic potency. |
|---|---|---|
| 81 | 0·1 | 2·63 |
| 82 | 1·0 | 1·92 |
| 83 | 10·0 | 4·7 |
| 84 | 100·0 | 9·7 |
| 85 | 1,000·0 | 14·4 |

The three lower dose levels produced responses which are hardly significant; the two higher doses both produced responses closely resembling that given by prolactin. At the highest dose level 34 per cent. of glands showed extensive glandular differentiation and the potency of this dose was approximately numerically equivalent to that of prolactin at one-tenth the quantity. Bearing in mind the possibility that this sample contains prolactin, we are inclined to account for the response obtained on this supposition, but the reaction merits further study.

### RESPONSE OF THE MALE WEANLING MAMMA TO GONADOTROPHIN

A standard preparation, " H.M.G.20 ", supplied by Messrs. Organon, was used for this experiment. It is prepared from pooled post-menopausal urine by absorption on kaolin and purification by tri-calcium phosphate. It possesses considerable gonadotrophic potency—1 mg. is derived from 15·67 ml. of urine. Three batches of 10 animals were used. They received, over a period of five days, the weight of hormone excreted over an increasing number of hours. The first batch injection represented 3 hours ; the second, 6 hours ; the third, 9 hours. The results are summarised in Table VIII.

TABLE VIII.

| Experiment number. | H.M.G. excretion. | Total glands. | Total clubs. | Mammotrophic potency. |
|---|---|---|---|---|
| 13 | 3 hours | 46 | 209 | 3·98 |
| 14 | 6 „ | 35 | 35 | 0·87 |
| 15 | 9 „ | 34 | 16 | 0·41 |

Within the limits of this experiment, the results show little or no response.

### RESPONSE OF THE MALE WEANLING MAMMA TO URINARY EXTRACTS

Our first series of experiments on the mammotrophic potency of extracts prepared from the urine of pre-menopausal women were for the most part disappointing. Vigorous growth responses were attained, but they were uncommon; a large number of extracts showed a low level of potency, and too many extracts produced negative results for no obvious reason. We suspected either that the Klinefelter technique instead of concentrating potency was, in fact, responsible for its loss (Hadfield, 1956), or that the output of the mammotrophic principle had a wide normal fluctuation during the menstrual cycle. The first clue emerged from a simple experiment, the significance of which may be clarified if the whole Klinefelter extraction process is summarised as follows :

Preparation of urinary extract by Klinefelter process:

STAGE I. 72 hours' specimen of urine. Sodium chloride (1 gm. to 100 c.c. added), then 4 vols absolute alcohol.

*Supernatant :*
Discarded.

*Deposit A :*
Largely composed of phosphates. Presumed to contain precipitated urinary and hormone proteins.

STAGE II. Deposit extracted with three successive aliquots of distilled water : centrifugalised.

*Supernatant :*
Presumed to contain all hormone protein in solution.

*Deposit B :*
Largely phosphatic : Presumed free of hormone protein. Discarded.

STAGE III. Dialysed at 0° C. against water. Protein re-precipitated by alcohol.

*Supernatant :*
Discarded

*Deposit C :*
(Still largely phosphatic. Presumed to contain all hormone protein.)

STAGE IV. Deposit dried, powdered and extracted with distilled water.

*Supernatant :*
Presumed to contain all hormone protein. Injected into animal.

*Deposit D :*
Presumed inactive. Discarded.

*Potency of deposits D and B*

It occurred to us that although the final phosphatic Deposit D is discarded on the assumption that it has no gonadotrophic potency, there was a possibility that it might retain mammotrophic potency. This supposition proved to be true, for when these deposits from twelve urines were suspended in distilled water and injected into twelve batches of mice they were found to be considerably more potent than their corresponding supernatants ; details are given in Table IX.

TABLE IX.

| Experiment number. | Mammotrophic potency of— Final Supernatant. | Deposit D. |
|---|---|---|
| 2 | 3·1 | 5·44 |
| 2 (Repeat) | 0·44 | 5·7 |
| 32 | 0·175 | 1·4 |
| 60 | 1·5 | 9·57 |
| 60 (Repeat) | 1·4 | 10·6 |
| 66 | 1·88 | 8·9 |
| 93 | 0·87 | 4·2 |
| 108 | 1·49 | 2·63 |
| 110 | 4·35 | 6·09 |
| 114 | 2·28 | 9·2 |
| 128 | 4·35 | 10·1 |
| 130 | 8·15 | 13·5 |

In this series, therefore, Deposit D was on the average three times more potent than its supernatant. There results led us to suppose that the heavy, previously discarded, first precipitate (Deposit B) may also be mammotrophic by retaining a proportion of the protein in the early stages of extraction. The following experiment not only confirmed this supposition but showed that this previously discarded deposit of some pre-menopausal urinary extracts had a mammotrophic potency of the same order as large doses of prolactin, and showed a similar capacity to induce a high degree of glandular differentiation in five days (Fig. 21, 22, 23). The results are shown in Table X, summarising a series of experiments in which suspensions in distilled water of Deposit B from four specimens of urine were injected into batches of mice at a dose level of 0·2 ml. twice daily for five days.

FIG. 10.

FIG. 11.

FIG. 10, 11.—Histograms showing distribution of clubs in glands treated with urinary extracts. 0 = No clubs. A = 1–5 ; B = 6–10 ; C = 11–15 ; D = 16–20 ; E = 21–25 ; F = 26–30 ; G = 31–35 ; H = 36–40 ; J = 41–45 ; K = 46–50 ; L = 51–55 ; M = 56–60. Fig. 10 : Deposit B from Experiment 120, over 5 days period. Fig. 11 : Deposit B from Experiment 132 over a 5 days period.

TABLE X.—*Potency of Deposit B.*

| Experiment No. | Number of mice. | Total glands. | Total clubs. | Mammo-trophic potency. | Percentage glands showing acinar formation. | Premature acinisation. |
|---|---|---|---|---|---|---|
| 120 | 13 | 60 | 1257 | 18·37 | 50 | ++ |
| 121 | 12 | 55 | 1110 | 17·7 | 55 | ++ |
| 132 | 19 | 97 | 1503 | 13·6 | 42 | ++ |
| 145 | 15 | 76 | 849 | 9·8 | 52 | ++ |

The protocols of Experiments 120, 121 and 132 are given below ; they demonstrate the general high level of mammotrophic potency possessed by phosphatic Deposit B and, in an equally striking manner, the great variation in reactivity of individual animals. This latter characteristic is not more marked than the irregular response of the mammae of individual weanling males to oestrone or prolactin. Histograms constructed from the results of Experiments 120 and 132 are shown in Fig. 10 and 11; the configuration of the latter resembles that produced by optimal doses of prolactin.

*Experiment No.* 120

A suspension in distilled water of one-fifth of the pooled previously discarded second Klinefelter deposits (Deposit B) from five 72 hours' specimens of urine.

| Mouse No. | Number of glands. | Club count. 1 | 2 | 3 | 4 | 5 | 6 | Total. |
|---|---|---|---|---|---|---|---|---|
| 1 | 5 | 18 | 8 | 5 | 2 | 23 | — | 56 |
| 2 | 4 | 58* | 18* | 22* | 48* | — | — | 146 |
| 3 | 4 | 32* | 30* | 20* | 37* | 17 | — | 136 |
| 4 | 6 | 18 | 19* | 12 | 12* | 16 | 24 | 101 |
| 5 | 5 | 21* | 6 | 5 | 5 | 14 | — | 51 |
| 6 | 6 | 11* | 24* | 7 | 40* | 13 | 18* | 113 |
| 7 | 5 | 8 | 20* | 25* | 15 | 13 | — | 81 |
| 8 | 6 | 46* | 34* | 46* | 23* | 32* | 47 | 228 |
| 9 | 5 | 10 | 11 | 5 | 11 | 13 | — | 50 |
| 10 | 3 | 0 | 3 | 0 | — | — | — | 3 |
| 11 | 3 | 28* | 5 | 33* | — | — | — | 66 |
| 12 | 2 | 42* | 60* | — | — | — | — | 102 |
| 13 | 6 | 32* | 4 | 43* | 22* | 6 | 17* | 124 |
| | 60 | | | | | | | 1257 |

T.C./T.G. = 1257/60. Mammotrophic potency = 18·37. 50 per cent of glands show acinar formation; of these, 60 per cent of branches are involved. Premature acinisation is common. (Glands showing acinar formation indicated *.)

*Experiment No.* 121

Previously discarded Klinefelter Deposit B from a 72 hours' specimen.

| Mouse No. | Number of glands. | Club count. 1 | 2 | 3 | 4 | 5 | 6 | Total. |
|---|---|---|---|---|---|---|---|---|
| 1 | 6 | 31* | 39* | 31* | 32* | 31* | 29* | 183 |
| 2 | 6 | 0 | 0 | 0 | 3 | 0 | 0 | 3 |
| 3 | 3 | 32* | 19* | 37* | — | — | — | 88 |
| 4 | 5 | 2 | 10 | 18 | 18 | 0 | — | 48 |
| 5 | 5 | 33* | 27* | 8* | 11 | 27* | — | 106 |
| 6 | 6 | 37* | 30* | 20* | 61* | 23* | 23* | 194 |
| 7 | 4 | 21 | 1 | 0 | 2 | — | — | 24 |
| 8 | 5 | 22* | 17 | 11 | 12 | 2 | — | 64 |
| 9 | 4 | 12* | 4 | 16 | 31* | — | — | 63 |
| 10 | 4 | 15* | 36* | 52* | 47 | 46 | — | 196 |
| 11 | 3 | 47* | 29* | 37* | — | — | — | 113 |
| 12 | 4 | 0 | 3 | 5 | 23 | — | — | 31 |
| | 55 | | | | | | | 1110 |

T.C./T.G. = 1110/55. Mammotrophic potency = 17·7. 55 per cent of glands show acinar formation; of these an average of 70 per cent of branches are involved. Premature acinisation is common. (Glands showing acinar formation indicated *.)

*Experiment No.* 132

Previously discarded Klinefelter Deposit B from a 72 hours' specimen of urine extending over the 13th, 14th and 15th days of the menstrual cycle.

| Mouse No. | Number of glands. | Club count. 1 | 2 | 3 | 4 | 5 | 6 | Total. |
|---|---|---|---|---|---|---|---|---|
| 1 | 5 | 9* | 1 | 3 | 21* | 3 | — | 37 |
| 2 | 5 | 10 | 4 | 10* | 12* | 12* | — | 48 |
| 3 | 5 | 11* | 1 | 4 | 18* | 22* | — | 56 |
| 4 | 6 | 10* | 13* | 6 | 4 | 11* | 11 | 55 |
| 5 | 6 | 21* | 8 | 14 | 8 | 8 | 3 | 62 |
| 6 | 6 | 33* | 22* | 5 | 44* | 22* | 16 | 142 |
| 7 | 5 | 11 | 17 | 4 | 27 | 4 | — | 63 |
| 8 | 6 | 25 | 45* | 37* | 42* | 37 | 30 | 216 |
| 9 | 6 | 32* | 27* | 10 | 24* | 35* | 37* | 165 |
| 10 | 5 | 0 | 5 | 2 | 8 | 8 | — | 23 |
| 11 | 6 | 19* | 14* | 10 | 10 | 4 | 10 | 67 |
| 12 | 5 | 19* | 31* | 17* | 16* | 14* | — | 97 |
| 13 | 6 | 16 | 0 | 10 | 5 | 16 | 2 | 49 |
| 14 | 4 | 51* | 47* | 48* | 18* | — | — | 164 |
| 15 | 4 | 10 | 2 | 12* | 9* | — | — | 33 |
| 16 | 4 | 24* | 30* | 5* | 12 | — | — | 71 |
| 17 | 4 | 0 | 2 | 5 | 0 | — | — | 7 |
| 18 | 5 | 31* | 26* | 14* | 30* | 13 | — | 114 |
| 19 | 4 | 18* | 2 | 8 | 6 | — | — | 34 |
| | 97 | | | | | | | 1503 |

T.C./T.G. = 1503/97. Mammotrophic potency = 13·6. 42 per cent of all glands show acinar formation ; of these, an average of 56 per cent of branches are involved. Premature acinisation is common. (Glands showing acinar formation indicated *.)

### POTENCY OF UNEXTRACTED URINE

The previously discarded phosphatic precipitate (Residue B) in the Klinefelter process probably carries a high proportion of the mammotrophic potency of the urine. The potency of such a residue prepared from a 72 hours' specimen (4500 ml.) may almost reach the potency and give rise to approximately the same degree of glandular differentiation as optimal doses of prolactin. In other words, the biological reaction produced by 4500 ml. of such a urine is *numerically* equivalent to a total dose of approximately 7500 $\mu$g. of prolactin, given over a period of five days, and 2 ml. would be equivalent to a total dose of 3·3 $\mu$g. of prolactin over the same period. This has a potency of about 15. It is reasonable to suppose, therefore, that normal unextracted and undiluted urine should produce a reaction of this order. Batches of ten mice were therefore given 0·2 ml. of untreated pre-menopausal urine twice daily for five days. The results of two experiments are shown in Table XI.

TABLE XI.

| Experiment No. | Number of mice. | Total glands. | Total clubs. | Mammotrophic potency. | Percentage glands showing acinar formation. | Premature acinisation. |
|---|---|---|---|---|---|---|
| 133 | 10 | 56 | 1078 | 16·9 | 60 | ++ |
| 153 | 10 | 47 | 508 | 9·4 | 32 | + |

The following is a protocol of Experiment No. 133.

*Normal urine* (not extracted).

| Mouse No. | Number glands. | Club count. 1 | 2 | 3 | 4 | 5 | 6 | Total. |
|---|---|---|---|---|---|---|---|---|
| 1 | 6 | 38* | 24* | 40* | 25* | 21* | 30* | 178 |
| 2 | 6 | 15* | 16* | 1 | 2 | 19* | 37 | 90 |
| 3 | 6 | 12* | 24* | 15* | 23* | 27* | 17* | 118 |
| 4 | 6 | 26* | 15* | 14* | 7* | 13* | 11* | 86 |
| 5 | 5 | 1 | 1 | 2 | 4 | 6 | — | 14 |
| 6 | 4 | 10* | 21* | 22* | 4* | — | — | 57 |
| 7 | 6 | 22* | 16 | 4 | 2 | 14 | 37* | 95 |
| 8 | 6 | 20* | 13 | 12* | 37* | 20* | 16* | 118 |
| 9 | 5 | 45* | 22 | 18* | 16* | 48 | — | 149 |
| 10 | 6 | 21* | 29* | 46* | 23* | 22 | 32* | 173 |
| | 56 | | | | | | | 1078 |

T.C./T.G. = 1078/56. Mammotrophic potency = 16·9. 60 per cent of glands show acinar formation; of these, 50 per cent of branches are affected. (Glands showing acinar formation indicated *.)

It is not justifiable to draw definite conclusions from these scanty observations and a number of untreated urines is now being examined. The results, however, are re-inforced by the fact that the potencies obtained are significant, they vary with the day of the menstrual cycle on which the specimens are collected, and the morphological changes in the reacting glands were characteristic, mature and immature glandular differentiation being well developed. If we are able to report that untreated urines possess significant degrees of potency, the clinical application of the method will clearly become a possibility.

### FLUCTUATIONS IN POTENCY DURING THE MENSTRUAL CYCLE

We have not been able to investigate this problem fully, but our results suggest that the cyclical fluctuations in potency are considerable. Our first experiment was conducted using the full Klinefelter extraction process and was carried out before we were convinced that this was responsible for loss of mammotrophic potency. Our figures are therefore proportionately lower than the actual values. The results are shown graphically in Fig. 12.

Disregarding the actual values, the significant rise in potency from the 16th to the 27th day is substantiated, using more potent extracts in a later but less complete study of the same individual. The tendency for potency to rise from an average figure of 8 on the 7th, 8th and 9th days to an average maximum of 15 on the 25th, 26th and 27th days has been borne out by eight separate estimations on other individuals at various phases of the cycle. We have some information, which needs further confirmation, that a sharp but unsustained rise of potency occurs at about the 16th day, that this is followed by a fall, and the fall by a rise reaching a peak about the 24th day.

As we are likely to use untreated urine in our future experiments, we would be inclined, in the case of pre-menopausal individuals, to select a specimen passed between the 19th and 27th days.

FIG. 12.—Graph showing fluctuations in mammotrophic potency during the menstrual cycle.

DISCUSSION

When the mammary response to the injection of pre-menopausal urine over a period of five days is compared with that produced by prolactin or oestrone over the same period there are no significant qualitative differences. There are, however, several quantitative differences. Urinary extracts and prolactin given twice daily for five days induce widespread glandular differentiation. Oestrone given over the same period at doses from 0·001 $\mu$g. to 0·01 $\mu$g. per day powerfully stimulates the growth phase and produces a fully developed duct system. It capacity to induce glandular differentiation over this period and at this dose level is significantly less than urinary extracts and prolactin. On the other hand, if low doses of oestrone are continued for eight to ten days an adequate growth phase is followed by normal widespread differentiation.

Under the influence of oestrone at higher dose levels, up to 32 $\mu$g. per day for five days, the degree of glandular differentiation produced approximates to that produced by prolactin and urinary extracts.

A striking feature of the response to oestrone at higher dose levels is that the growth phase appears to be arrested in many glands before their duct systems are fully grown and widespread differentiation takes place in partly grown glands. " Premature acinisation " in glands which before stimulation had a very simple structure is characteristic of the response to urinary extracts and to prolactin.

These considerations suggest that full mammogenesis is a two-phase process which depends on the synergistic action of two hormones and we would venture to speculate that oestrone may be responsible for the growth phase and prolactin for the phase of glandular differentiation.

The experiments of Lyons (1943) and of Lyons and his collaborators (1952 ; 1955), using gonadectomised and hypophysectomised immature male and female rats, show that the maximum growth and differentiation of their mammary glands demands the synergistic action of prolactin and oestrone acting in optimal proportions, prolactin acting at a far higher level of dosage than oestrone. The interpretation of our results would become easier if any proof were forthcoming that oestrone is capable of producing direct or indirect stimulation of the hypophysis with the liberation of prolactin.

The urine of normal pre-menopausal women may therefore be mammotrophic to immature male mice not because it contains either prolactin or biologically active oestrogens but by virtue of the fact that it contains both these hormones. On this supposition, its potency may depend not only on the presence of these two hormones but also upon their relative concentrations, and the experiments of Lyons and his colleagues suggest that the prolactin concentration must be high whilst that of active oestrogen may be surprisingly low.

### CONCLUSION

Using groups of ten weanling male mice yielding a total of at least 48 mammary glands and regarding the average number of clubs per gland as a measure of the mammotrophic potency of an injected urinary extract, we would claim, on the basis of 54 examinations of urinary extracts and untreated urines, to have confirmed the suggestion made in 1955 that the urine of normal pre-menopausal women contains a biologically active substance, or substances, which stimulates growth and glandular differentiation in the rudimentary mammae of immature males.

We are indebted to Dr. J. W. Trevan, F.R.S., for the statistical analysis and would like to thank Dr. A. T. Cowie for supplying us with prolactin and Dr. A. L. Greenbaum for the growth hormone.

We have received valuable assistance throughout from Dr. R. D. Bulbrook and Dr. F. C. Greenwood on biochemical matters, and this work could not have been accomplished without the willing co-operation of the laboratory staff and of the Photographic Department of the Fund's laboratories at Lincoln's Inn Field.

### REFERENCES

Hadfield, G.—(1956) *Brit. med. J.*, **1**, 94.

Klinefelter, H. F., Junr., Albright, F. and Griswold, G. C.—(1943) *J. clin. Endocrin.*, **3**, 529.

Lyons, W. R.—(1943) ' Essays in Biology '. Berkeley (University of California).

*Idem*, Johnson, R. E., Cole, R. D. and Li, C. H.—(1955) ' Hypophyseal Growth Hormone, Nature and Actions : International Symposium '. New York (McGraw-Hill Book Co. Inc.), p. 461.

*Idem*, Li, C. H. and Johnson, R. E.—(1952) *J. clin. Endocrin.*, **12**, 15.

Scowen, E. F. and Hadfield, G.—(1955) *Cancer*, **8**, 890.

### EXPLANATION OF PLATES.

FIG. 13.—Colour photograph showing the skin, deep surface uppermost, after fixation in Bouin's fluid and 24 hours in 50 per cent alcohol. The landmarks will be found by reference to Fig. 1 in the text.

FIG. 14.—The undissected skin has been stained in Grenacher's alum carmine. The position of the glands is shown in a tracing of the photograph (Fig. 1 in text).

FIG. 15.—Fourteen normal glands mounted whole and unsectioned, showing normal structural variation in the strain of mice used for these experiments. The first ten glands are unclubbed and rudimentary ; the last four bear clubs. × 25.

FIG. 16.—Effect of oestrone. A total of 10·53 $\mu$g. was given over a period of 5 days. Both glands are in the growth phase. Deeply-stained club-bearing buds are common. A few branches show early differentiation but the outline in the majority is sharply defined. × 25.

FIG. 17.—Photomicrographs showing growth phase induced by oestrone. A. Shows acellular and almost solid club. Differentiation has not yet commenced. × 200. B. Cellular detail of above. There is lack of cellular differentiation and evidence of proliferation. × 450.

FIG. 18.—Photomicrographs showing cytological changes in terminal clubs induced by oestrone. × 1000. A. Several mitotic figures are visible. The cells are undifferentiated and have active nuclei. There is evidence of early glandular differentiation. B. The same changes are visible on the left and the mesenchymatous reaction at the edge of the club is seen on the right.

FIG. 19.—Photomicrograph showing cytological changes produced by prolactin. (Total dose 5000 $\mu$g. over a period of 5 days.) A. Cellular proliferation in a club, also acinar differentiation. × 450. B. Early glandular differentiation and nuclear detail. ×1000.

FIG. 20.—Effect of prolactin : Total dose = 10,000 $\mu$g. : duration 5 days. The upper part of the gland is still in the growth phase. Below this clubs show progressive decrease in size. Glandular differentiation is well established in the lower part of the gland. × 25.

FIG. 21.—Effect of urinary extract with a potency of 17·7. Injection period = 5 days. A few clubs are still present in the upper left corner ; below this glandular differentiation is well established. × 25.

FIG. 22.—Photomicrographs showing the cytological response to the injection of urinary extract twice daily for 5 days. Three cellular clubs lying in reacting mesenchyme showing progressive acinisation whilst growth phase is still active. × 450.

FIG. 23.—Photomicrographs showing glandular differentiation produced by injection of urinary extract twice daily for 5 days. A. A dilated duct from which project series of partially hollowed-out solid buds. × 200. B. Part of A, showing the cellular buds to be primitive glandular acini. × 450.